# Increased shark bite survivability revealed by two centuries of Australian records

**DOI:** 10.1038/s41598-022-16950-5

**Published:** 2022-08-19

**Authors:** James P. Tucker, Isaac R. Santos, Brendan P. Kelaher, Marcel Green, Graeme F. Clark, Paul A. Butcher

**Affiliations:** 1 National Marine Science Centre, Southern Cross University, Coffs Harbour, NSW Australia; 2 Department of Marine Sciences, University of Gothenburg, Gothenburg, Sweden; 3 Department of Primary Industries, New South Wales, Sydney Institute of Marine Science, Mosman, NSW Australia; 4 School of Biological, Earth and Environmental Sciences, University of New South Wales, Sydney, Australia; 5 New South Wales Department of Primary Industries, National Marine Science Centre, Southern Cross University, Coffs Harbour, NSW Australia

**Keywords:** Marine biology, Animal behaviour

## Abstract

The perceived and real threat of shark bites have significant direct health and indirect economic impacts. Here we assess the changing odds of surviving an unprovoked shark bite using 200 years of Australian records. Bite survivability rates for bull (*Carcharhinus leucas*), tiger (*Galeocerdo cuvier*) and white (*Carcharodon carcharias*) sharks were assessed relative to environmental and anthropogenic factors. Survivability of unprovoked bull, tiger and white shark bites were 62, 75 and 53% respectively. Bull shark survivability increased over time between 1807 and 2018. Survivability decreased for both tiger and white sharks when the person was doing an in water activity, such as swimming or diving. Not unsurprisingly, a watercraft for protection/floatation increased survivability to 92% from 30%, and 88% from 45%, for tiger and white sharks respectively. We speculate that survival may be related to time between injury and treatment, indicating the importance of rapid and appropriate medical care. Understanding the predictors of unprovoked bites, as well as survivability (year and water activity), may be useful for developing strategies that reduce the number of serious or fatal human-shark interactions without impacting sharks and other marine wildlife.

## Introduction

Following the release of the movie *Jaws* in 1975*,* the profile of sharks as so-called ‘man eaters’ increased dramatically^1^. This perception has been further exacerbated by modern sensationalist media reports referring to human-shark interactions using terms such as ‘rogue shark’^2^ and ‘man eater’^3^ and as ‘stalking’ and ‘lurking’ beyond the surf break just waiting for humans/the next meal to arrive. However, the probability of a shark-human interaction is extremely low and the likelihood of a bite being fatal is even lower^4^.

**Figure 1:**
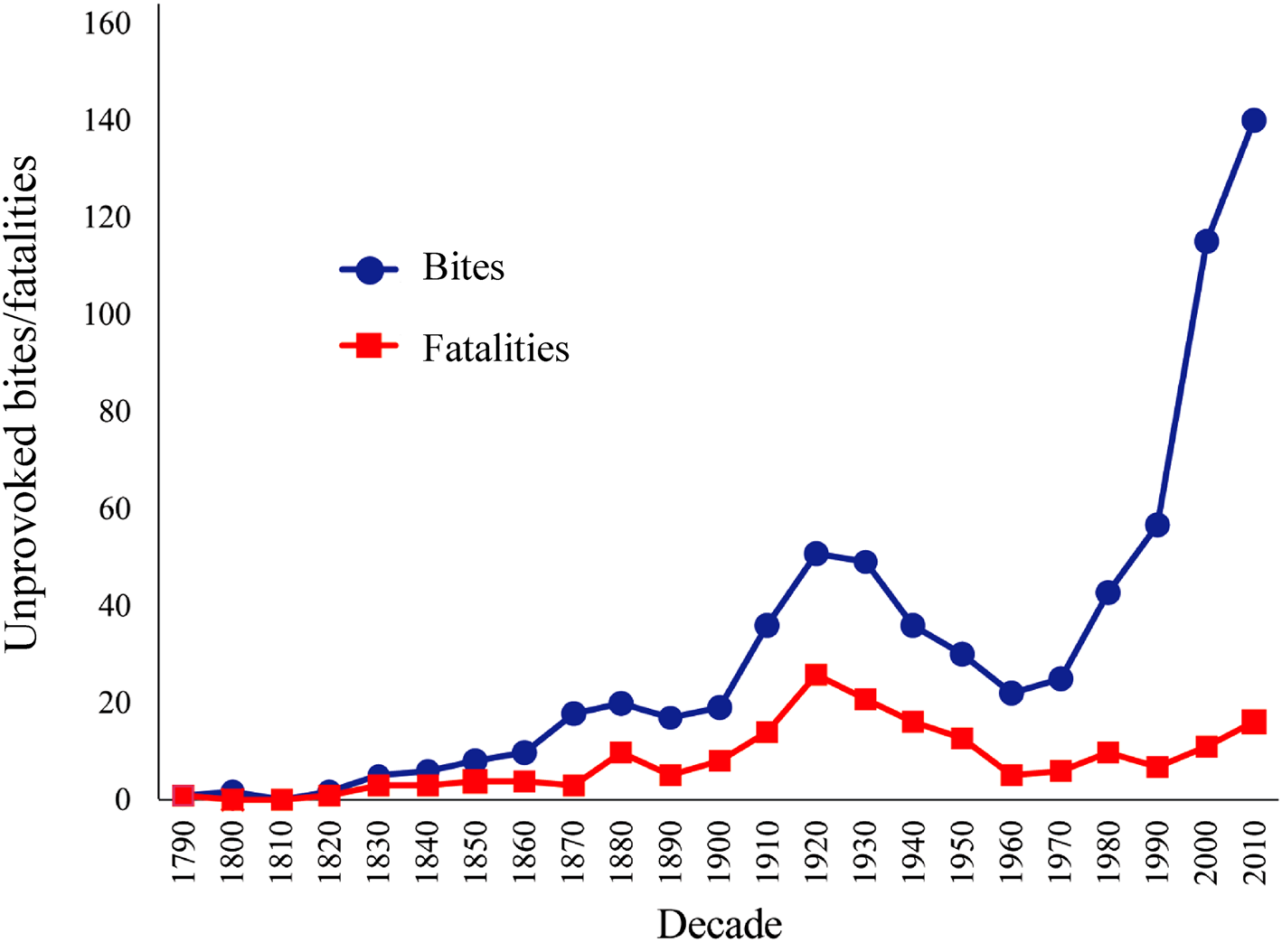
Annual frequency of recorded unprovoked shark bites and fatalities in Australia 1790–2018.

While the likelihood of being bitten or killed by a shark is small, the effects on the people involved and associated communities are significant, often resulting in negative attitudes or perceptions towards sharks^5^. The complex relationship between sharks and humans has an influence on shark protection and management. Mitigation of shark bites includes destructive techniques, such as meshing programs and baited drum lines^6^. These mitigation techniques increase pressure on species that are threatened, like greynurse (*Carcharias taurus*) and white (*Carcharodon carcharias*) sharks, as well as other marine life impacted as bycatch^7^. Sharks often play an important ecological role and are commonly apex predators in their ecosystems^8,9^, so destructive mitigation programs have negative effects on shark populations, non-target animals and associated ecosystem processes (Fig. 1).

**Figure 2:**
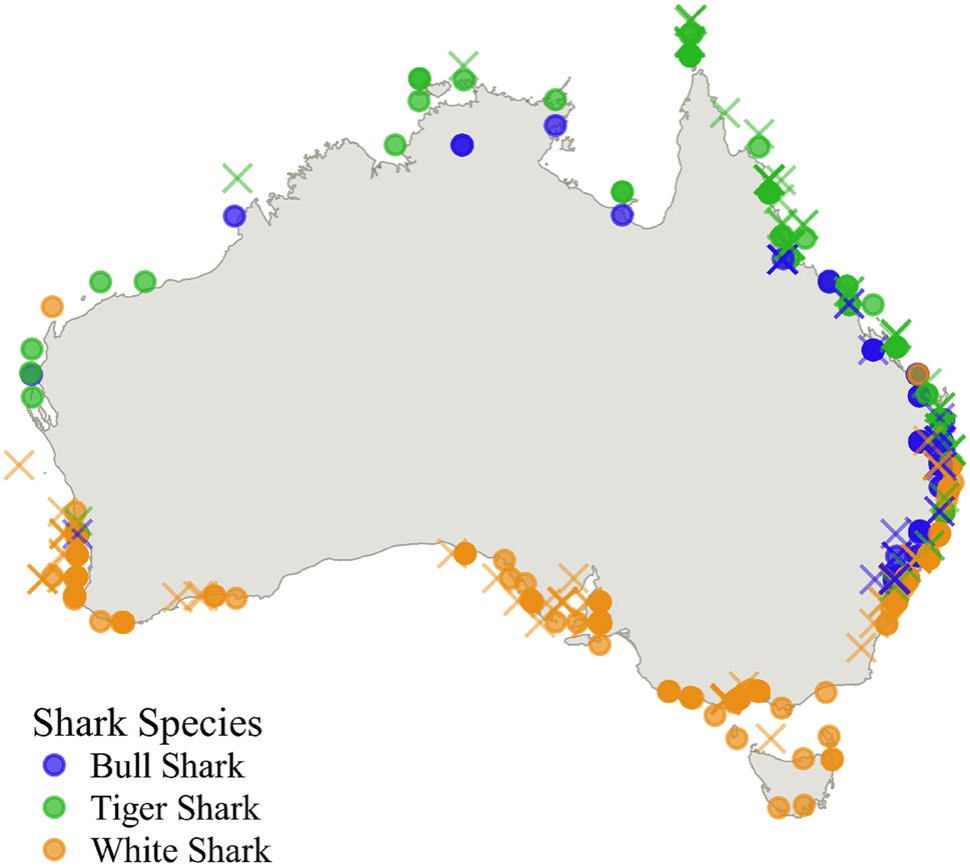
Location of Australian shark bites 1790–2018. Circles represent survived bites, X represents fatalities. Figure created using QGIS v. 3.16.2 (www.qgis.org).

The occurrence of unprovoked shark bites has increased globally for over 30 years and Australia has been identified as a ‘hotspot’^10^. Records of shark bites have been maintained in Australia since 1791. More recently, additional data fields have been collected for each event, such as habitat type, species of shark, activity of person(s) involved and injuries, enabling a more comprehensive assessment of events (Fig. 2). The probability of surviving a shark bite is an important factor when assessing the potential risk of shark-human interactions. While most investigations have quantified temporal trends in shark bites^11–13^, they do not provide specific detail on the potential drivers of human survivability.

The data documenting Australia’s history of shark bites has been used to assess the frequency^12^ and environmental predictors of bites^11^. Here, we build on these earlier analyses by assessing the probability of human survivability of unprovoked shark bites. We focus on the three species considered most dangerous to humans in Australia; bull (*Carcharhinus leucas*), tiger (*Galeocerdo cuvier*), and white (*Carcharodon carcharias*) sharks. We examined the influence of environmental (temperature, season, and habitat) and human (population density, present mitigation strategies, injury location on body, and water activity) factors on survivability of shark bites. Understanding these key factors that increase the survivability of shark bites supports the development of both evidence-based mitigation strategies to reduce the frequency of interactions, and post-bite management to reduce the impact of human-shark interactions.

## Methods

Data was sourced from—Australian Shark Incident Database (ASID), Taronga Conservation Society Australia (https://www.taronga.org.au/conservation-and-science/australian-shark-incident-database). Data was provided with permissions from Taronga Conservation Society Australia to analyse for research purposes. Shark species involved in an bite were identified using bite analysis and artifacts from the injury site (such as teeth). Here, we focused on survivability of all unprovoked events up to, and including, 2018 for bull, tiger and white sharks. We defined an unprovoked bite as an interaction wherein the person(s) was injured or knocked from their unpowered watercraft. Interactions with powered watercraft and events when sharks ‘bumped/nudged’ unpowered watercrafts were not included. Water activities being undertaken by a person were separated into two groups; ‘on water’ and ‘in water’. Human population data was taken from the Australian Bureau of Statistics (https://www.abs.gov.au/).

In order to assess potential drivers of shark bite survivability, statistical analyses were done in R v. 3.5.0 (www.r-project.org). For each species, a generalised linear model (GLM) was used to estimate the probability of surviving an bite as a function of the following predictor variables: year, habitat (as defined by Australian Shark Incident Database), season, meshed/protected area (area protected by exclusion barrier, including netted ocean pool), distance from river, latitude, activity (activity person was performing at the time of bite, such as surfing, swimming, diving, etc.), injury location (on human body), person’s age and shark size. Prior to modelling, correlations between predictors were assessed to ensure none exceeded 0.6. Sea-surface temperature was removed due to correlation with latitude, and human population was removed due to correlation with year.

Backwards stepwise model selection was used, starting with a full model and sequentially removing terms that significantly decreased Akaike information criteria (AIC) when removed (Zuur et al., 2009). For significance tests during model selection, we used loglikehood ratio tests with χ2‐distribution, implemented in the ‘drop1’ function (Chambers, 1992). The DHARMa package was used for diagnostics of final models, which were inspected with quantile‐quantile (Q‐Q) plots, residuals v. fitted values plots and residuals v. predictors plots^14^. Formal tests for goodness of fit, overdispersion, zero inflation, and temporal autocorrelation were found to be non-significant for each of the three shark species^14^.

## Results

**Figure 3:**
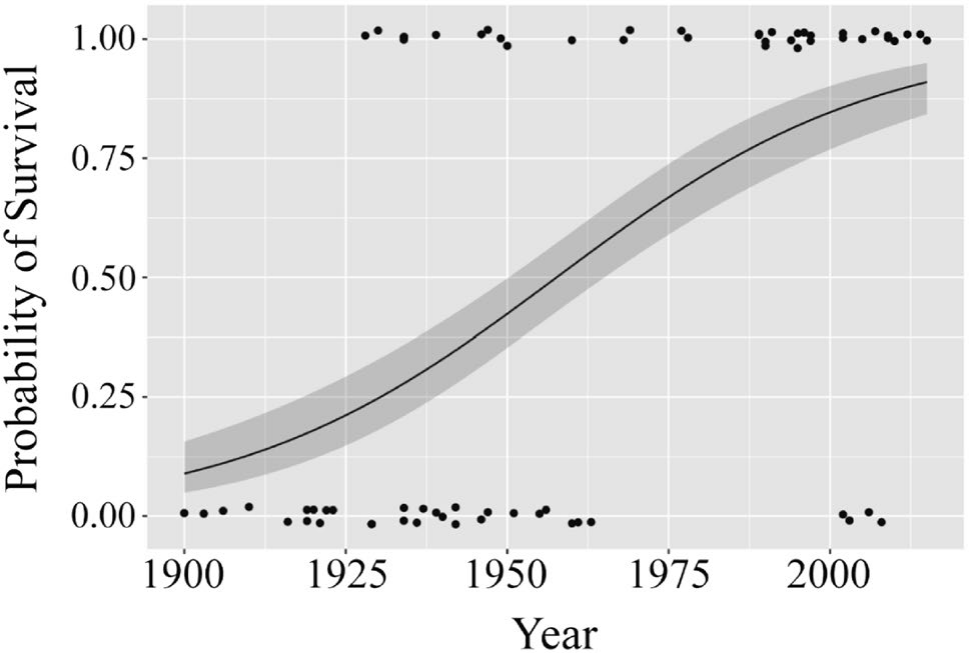
Probability of surviving a bull shark bite over time. Time was not a significant predictor for survivability of tiger and white bites. Figure created using R v. 3.5.0 (www.r-project.org).

**Figure 4:**
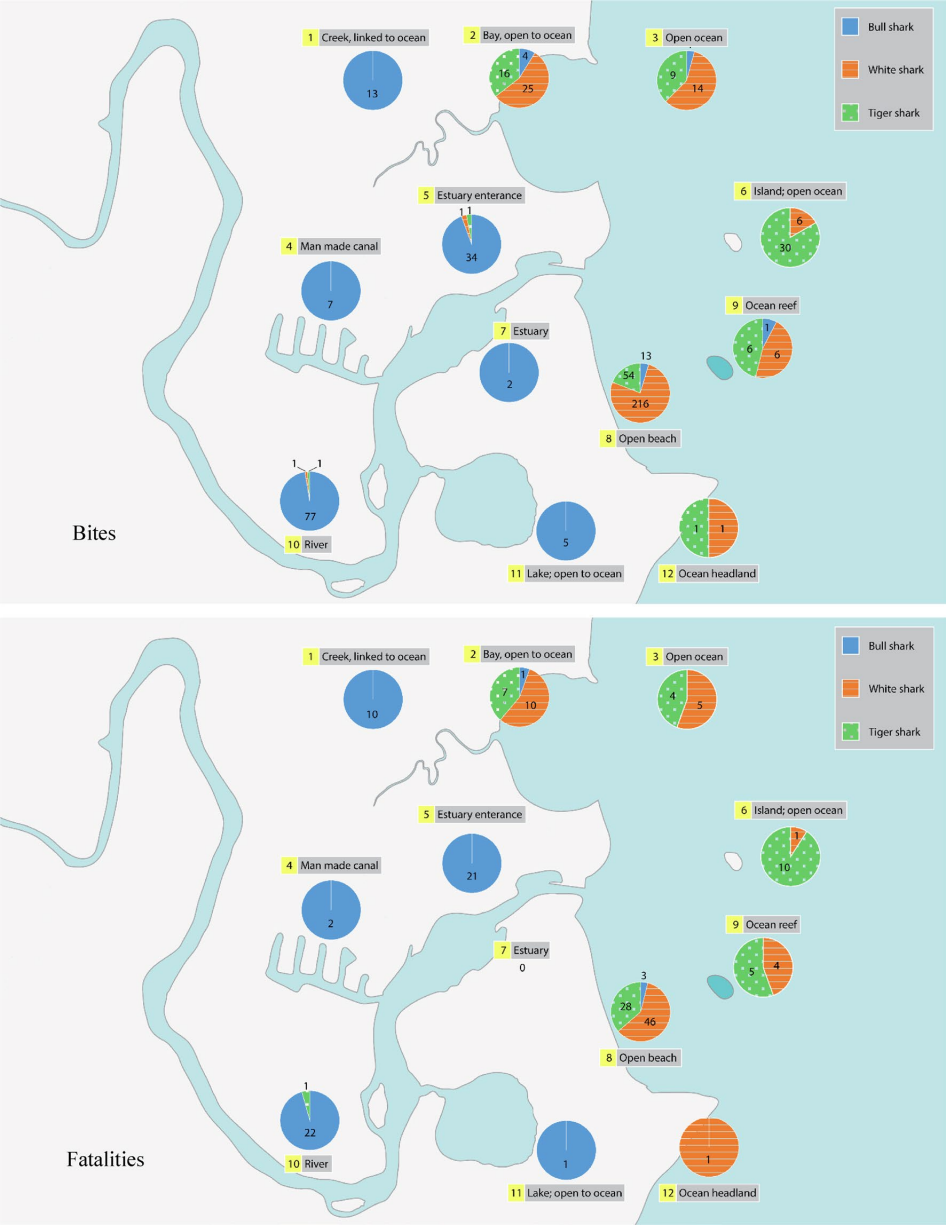
Bites and fatalities across habitats for bull, tiger and white sharks 1791–2018. Figure created using QGIS v. 3.16.2 (www.qgis.org).

There were 157, 120 and 270 bull, tiger, and white shark bites, respectively, included in the analysis. Of these bites 60, 56 and 67 were fatal for bull, tiger, and white sharks respectively. The percentage survivability of unprovoked bull, tiger and white shark bites were 62, 53 and 75% respectively. Of the nine predictor variables explored only three had significant explanatory power. Year was a significant predictor for surviving bull shark bites (*p* < 0.001), with the survivability rate increasing over time (Fig. 3). Location and habitat type influenced bites for each of the three species, although the trends did not directly correlate to survivability. Eighty-six per cent of bull shark bites occurred in estuarine and freshwater systems (Fig. 4), but only 2% of tiger shark and 0.4% of white shark bites were recorded in these habitats. Sixty-seven per cent of tiger shark bites occurred at open beaches and islands in the open ocean (Fig. 4). Ninety-four per cent of white shark bites occurred at open beaches, in the open ocean and in oceanic bays (Fig. 4).

**Figure 5:**
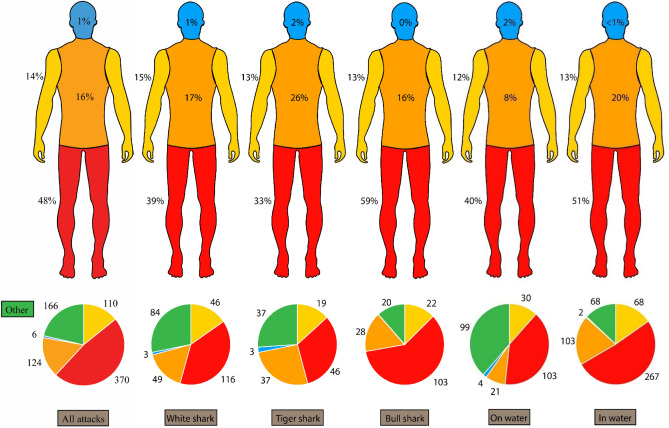
Percentage of injury locations (on human body) (**a**) across shark species, (**b**) across water activity type. Colours in pie charts match body part colours. Other (green) includes data with no injury recorded.

**Figure 6:**
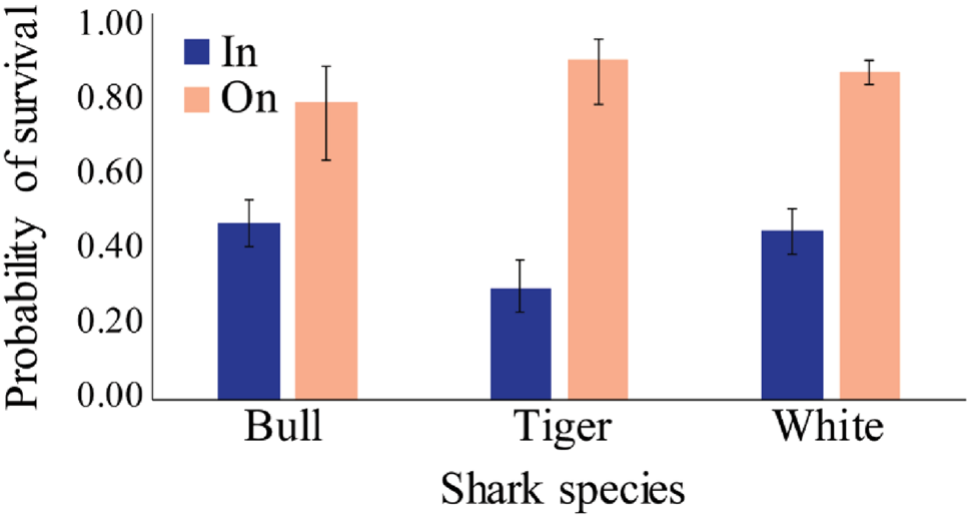
Survivability of shark bites for in water vs on water activities for bull, tiger and white sharks. On water activities include being on any unpowered vessel; surfing, bodyboard, kite board, kite surf, stand up paddle board, canoe, floatation device, kayak, rowboat, surf ski, wake boarding. In water activities include fishing (standing in water), free diving, SCUBA diving, snorkelling, standing/wading, swimming, swimming with unpowered vessel (e.g. clinging to wreckage).

Legs are the most commonly injured human body part in shark bites (Fig. 5), with 40% for on water and 51% for in water activities. There was more variation in injury type for in water activities (Fig. 5). The strongest predictor for survivability of a tiger or white shark bite is the type of activity (*p* < 0.01 and *p* < 0.001 respectively; Fig. 6). Chances of shark bite survival also significantly increased when a person is undertaking an on water activity (such as surfing or kayaking), with 92% and 88% chance of survival for tiger and white sharks respectively, compared to 30% and 45% chance of survival during in water (such as swimming for SCUBA diving) activities for tiger and white sharks respectively.

## Discussion

The probability of surviving an unprovoked bull shark bite has increased over years likely due to faster emergency response, advances in medicine and first aid, increased number of water users being nearby to assist a person who has been bitten, and changes in water user behaviour. Caldicott, et al.^15^ attributes deaths from shark bites to a lack of on-scene resuscitation, haemorrhagic shock or drowning. Woolgar, et al.^16^ attributes 8 out of the 10 fatalities in their review to exsanguinating haemorrhage from limb vascular injuries. The general treatment of severe trauma has improved over time, so that injuries that were once fatal can now be more effectively treated^17,18^.

The time between suffering an injury and stopping a haemorrhage has been recognised as crucial in affecting survival outcome^19,20^. This is supported by bull shark survivability. Bites that occur inland (in rivers and estuaries), or closer to the shore, may result in faster response times and thus increase survivability. Training in basic first aid, and the quality of training, has increased over time^21^, increasing the chance that a person involved in a shark bite can receive rapid treatment. In Australia, Surf Life Saving club members present across most urban beaches receive first aid training^21^, improving the chance of bite victim survival. Nonetheless, severe injury survivability generally requires the victim to receive professional medical care in a timely manner^22^.

The timeliness and quality of shark bite reporting has improved over time which may have influenced the apparent increased survivability rate in recent decades. With the growth of technology and particularly social media, major and minor interactions with sharks are more likely to be recorded. The shark incident database indicates that recent bites, especially minor ones involving generally harmless Wobbegong Sharks (not included in our analysis), have been added due to their appearance in the media^23^. Therefore, it is likely that only significant earlier shark bites received public attention and database inclusion. This may have biased the trend in bull shark survivability over time as less serious bites may not have been reported by victims in earlier years, or were reported but not yet uncovered by the ASID investigators, decreasing our overall estimate of survivability in these earlier years. Increases in scientific reporting can also be driven by more publicly available and/or increased public awareness of official records. This has occurred in fisheries science^24^ with reporting biases now well understood^25–27^.

The apparent increase in bites after 1990 (Fig. 1) is likely to be partly due to increased capacity for and almost instantaneous nature of public reporting. This is supported by the number of fatalities remaining relatively stable while total bites have increased. Due to their seriousness, reports of any fatalities are likely to be entered in the records in any decade while bites with no or minor injuries may not have been reported as frequently before the 1990s. This is borne out to some degree using Wobbegong bites as an example. In the period from 1900 to 1999, there were 15 minor bites attributed to Wobbegongs in the State of New South Wales, but since 2000 there have been 38 reports of Wobbegong bites. The increase in the popularity of water activities, particularly in and around inshore reefs frequented by Wobbegong Sharks and recovery of shark populations temporally^13^, may also have influenced this trend.

Each species displayed characteristic patterns in the geographic range and habitat of bites. Most bull shark bites occurred in freshwater and estuarine systems, particularly in the Sydney area in the late 1800s and early to mid-1900s. Bathing in the waters of Sydney Harbour over summer was a popular pastime in the early 1900s, despite the poor water quality due to the factories and processing works releasing untreated effluent. Of the 12 fatalities attributed to bull sharks in NSW, only one occurred at an ocean beach (adjacent to the mouth of the Richmond River after rainfall), with the other 11 occurring in Sydney estuaries between 1900 and 1963 and all in the austral summer months of December and January. The number of reported bites and fatalities attributable to bull sharks is also likely to be markedly underestimated, as there were at least 15 fatalities in Sydney and Newcastle estuaries between 1900 and 1960 during the austral summers that are attributed to ‘unidentified species’ or ‘whaler species’, that based on our current knowledge of the movements of bull sharks strongly indicates that were more likely to be responsible for the majority, if not all, of those bites.

While bull sharks range from these lower salinity environments to oceanic waters, tiger and white sharks are primarily found in higher salinity oceanic waters. Tiger shark bites dominate open ocean and islands which may be explained by Australia’s geography. There are more islands in northern Australia due to the presence of coral reefs^28^ and tiger sharks are more commonly found in warmer, northern, waters^29,30^, although they also inhabit cooler temperature conditions^31^. In contrast, white sharks are commonly found in colder waters in the mid to high latitudes^32,33^. Open beaches, open ocean and ocean bays, as well as large and ever increasing population centres, are common at these latitudes in Australia^34^. The increasing popularity and mainstream nature of modern surfing has also seen a marked increase in bites on surfers. Up until the 1960s, almost all white shark bites were on swimmers, mostly fatal, whereas since the 1960s most of the bites attributed to white sharks have been on surfers. This is likely due to a number of factors, including that swimmers are generally protected by surf life savers between the flags, the increasing number of surfers, the improvements in wetsuit technology that allow surfers to surf all year round, and the propensity to surf isolated breaks and/or alone. Similar changes in trends have been observed more recently in Brazil when there were changes in dominant water activities^35^. In Pernambuco, Brazil, surfing was prohibited for a period when the water activity involved in the majority of bites changed from relatively equal parts swimmers and surfers (before prohibition of surfing) to majority swimmers (after prohibition)^35^.

The body part(s) of a person can also play a role in survivability. Not unsurprisingly, legs are the most commonly injured limb as they suspend below unpowered watercrafts and are moving during in water activities, possibly raising the interest of nearby sharks. Sharks are known to use test biting to investigate potential prey and, while this is not considered a consumptive behaviour, the location of the femoral artery means that injuries can be serious and may lead to fatality^36^. Legs and thighs are also often bitten because of the bite mode and behaviour of the shark, which often appear to have struck from the side or underneath, indicating some innate behavioural strategy. Legs are also often used defensively, increasing the chances of an injury. There is more variation in injury location during in water activity suggesting that ‘access’ to these body parts played a role in where injuries occurred and, therefore, water activity may influence injury location. Similar trends in body part injuries and water activity have been observed in other parts of the world^13^.

Survivability of tiger and white shark bites decreased when a person was conducting an in water activity. Between 1950 and 2003, white shark bites in the USA showed similar trends with 9.5% of bites being fatal, and 77.8% of fatal bites occurring on people conducting in water activities^37^. On water activities often rely on a watercraft that can be used for protection and/or to reach the shore quickly, reducing the treatment time and possibly the injury extent. Having a floatation device may also reduce the risk of drowning after sustaining serious injuries. Finally, the visibility of an bite by bystanders may be increased with on water activities, which could decrease the time between an bite and first aid. Some in water activities, such as scuba diving, may be more difficult to observe from the surface or shore, and may also be influenced by water visibility and depth.

The severity of a shark bite may differ with different activity types. A shark biting a watercraft is considered an unprovoked bite, however, there is commonly no physical injury to a person which directly relates to survivability. There are many instances of shark bites during water activities where a board/floatation device is severely damaged, but the person is unharmed or suffers minor injuries^38^. During an in water activity, the lack of large equipment for protection means that a test bite/bite is likely directed at the person, causing injury and decreasing the chance of survivability.

Understanding and communicating the drivers of shark bites is an important part of mitigating human-shark interaction. Shark mitigation focuses primarily on reducing the number of negative human-shark interactions^39^. The result of an interaction, including injury severity and person’s survivability, should also be considered by government agencies and beach authorities when determining appropriate post-interaction responses. The trends of survivability with the type of water activity should also be considered in future mitigation plans. Information packages provided to stakeholders should include the predictors of survivability which may influence the type of activities water users undertake, and of the importance of rapid and effective trauma management. More beach goers, particularly surfers, are becoming familiar with the correct use of tourniquets and minimising blood loss, and authorities should consider the distribution or placement of trauma kits in key locations.

Historically, mitigation to reduce human-shark interactions in Australia has relied on baited drumlines, meshing programs and estuarine swimming enclosures^7,39,40^. While these methods may be effective to some degree, the drumlines and mesh nets also impact shark populations and other marine wildlife, which includes threatened and protected species^40^. Although the rate of bites has reportedly been reduced at the beaches protected by these mitigation techniques^41^, survivability of tiger and white shark bites did not directly correlate with timing of mitigation interventions, nor did they provide any broader scale reduction in the rate of interactions or completely stop bites at meshed beaches^42^. There have been over 30 shark bites at NSW meshed beaches including a few serious injuries. If those events had not occurred near areas with rapid first aid and proximity to world class hospitals, they could have been fatal. Estuarine swimming enclosures and messaging around ‘not swimming at dawn and dusk’ appear to be at least partly responsible for the reduction in bull shark interactions, particularly near Sydney^43^, but those strategies have not reduced the number or severity of interactions with tiger or white sharks. There is a clear need to implement new mitigation techniques that are more specific to both surfers and white sharks.

## Conclusions

We used a historical dataset spanning two centuries to reveal that: (1) the survivability of bull shark bites has increased over time, and (2), that survivability of tiger and white shark bites increases when people are conducting on water activities such as surfing and kayaking. The aim of modern shark bite mitigation is to reduce the frequency and severity of human-shark interactions, while preventing the destruction of sharks and/or other marine life. Understanding trends in historic shark bites helps to recognise drivers of these interactions and indicates that it is time to reassess shark mitigation programs used in Australia, and potentially elsewhere.

## Supplementary Information

Supplementary Information 1.

Supplementary Information 2.

Supplementary Information 3.
