# Supplementary material for: Increased shark bite survivability revealed by two centuries of Australian records
Source: Sci Rep. 2022 Aug 19;12:14121. doi: 10.1038/s41598-022-16950-5 (PMC9391475; doi:10.1038/s41598-022-16950-5)

BULL SHARKS

- Load data and libraries

```
dd <- read.csv("SharkDeaths.csv")
library(DHARMa)
library(ggplot2)
library(GGally)
library(boot)
library(visdat)
```

- add Survival
- subset for the species

```
dd$Survival <- 1 - dd$Death
bull <- dd[dd$Species=="BS",]
```

- inspect missing data and correlations between predictor variables

```
vis_miss(bull)
```

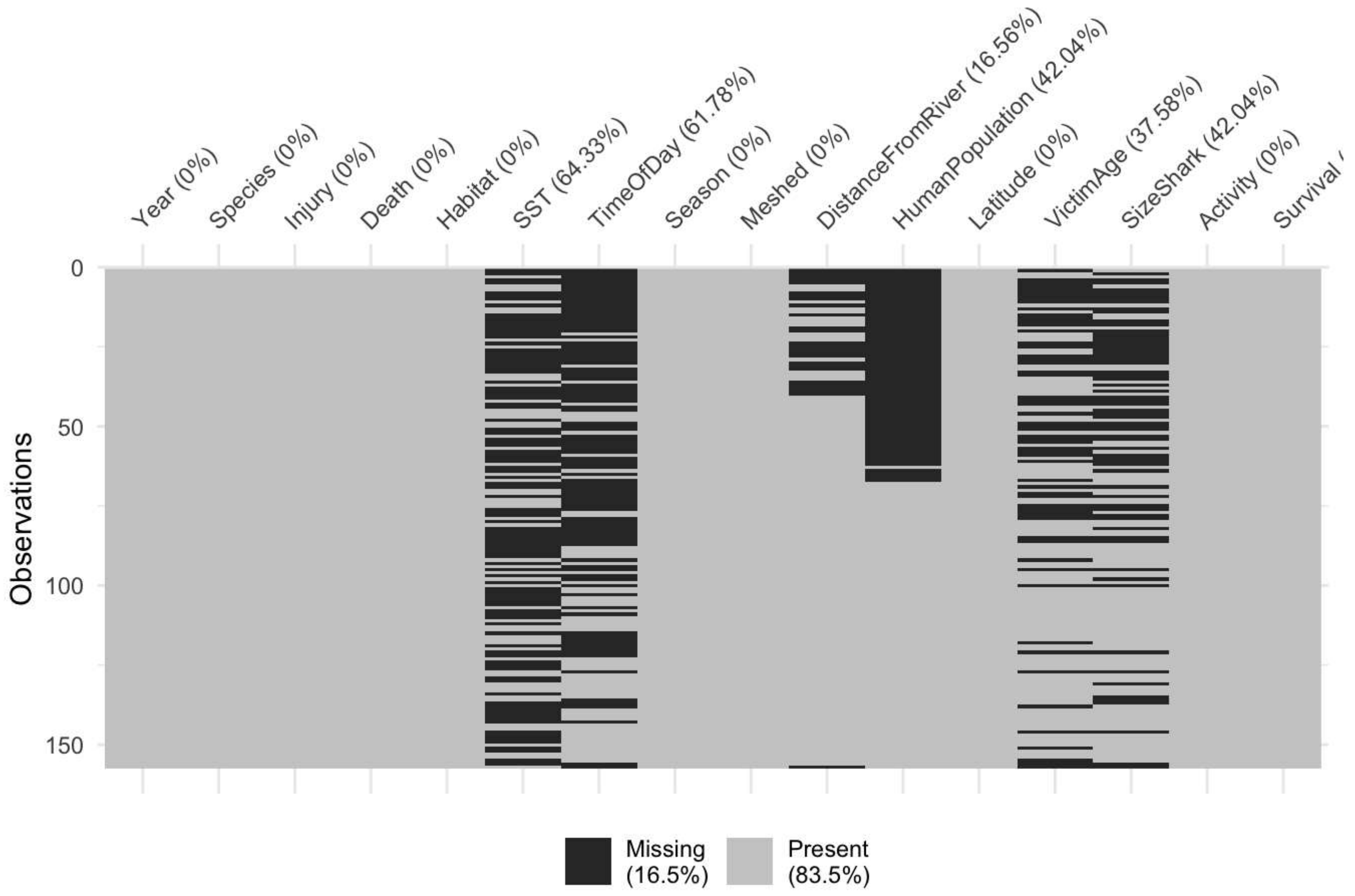


```
numericPredictors <- names(bull)[names(bull) %in%
                        c("Habitat","Species","Season","Activity") == F]
ggpairs(bull[,numericPredictors])
```

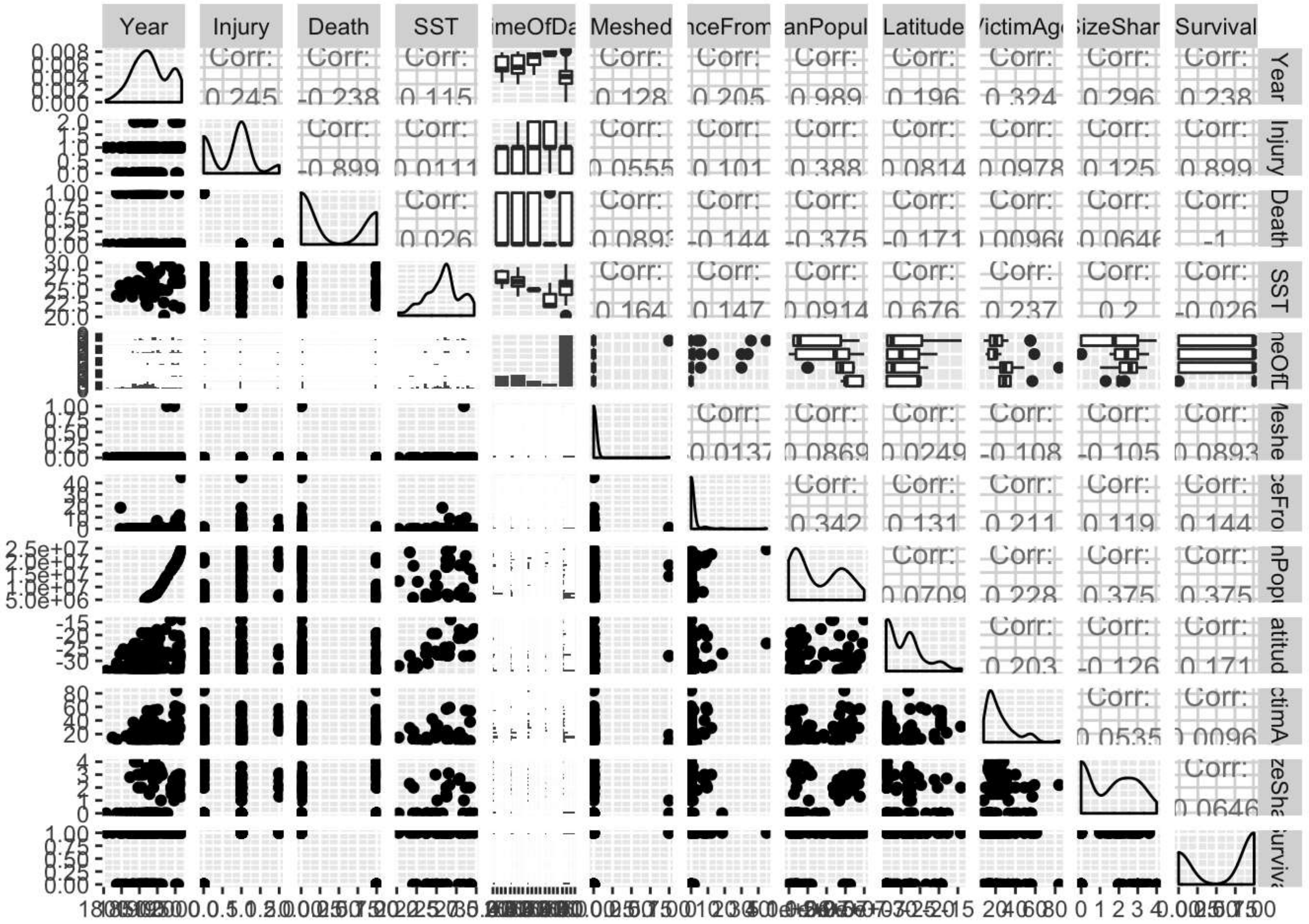

- Remove SST (missing data and correlated with Latitude)
- Remove Human Population (missing data and correlated with Year)
- Remove Injury (not of interest)
- Remove TimeOfDay (missing data and not of interest)

```
bull$ HumanPopulation <- NULL
bull$ Injury <- NULL
bull$ SST <- NULL
bull$ TimeOfDay <- NULL

numericPredictors <- names(bull)[names(bull) %in%
                        c("Habitat","Species","Season","Activity") == F]
ggpairs(bull[,numericPredictors])
```

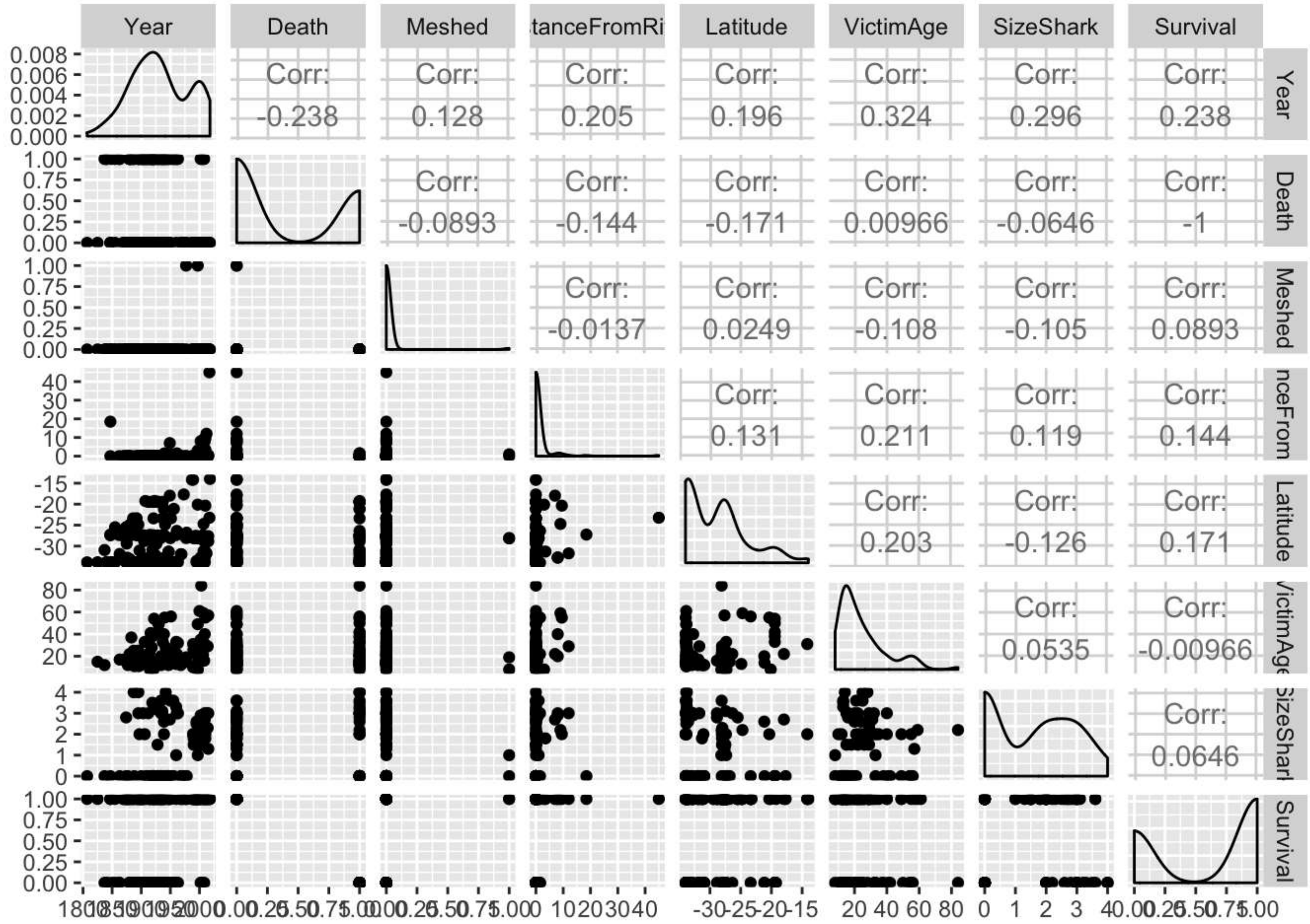


```
vis_miss(bull)
```

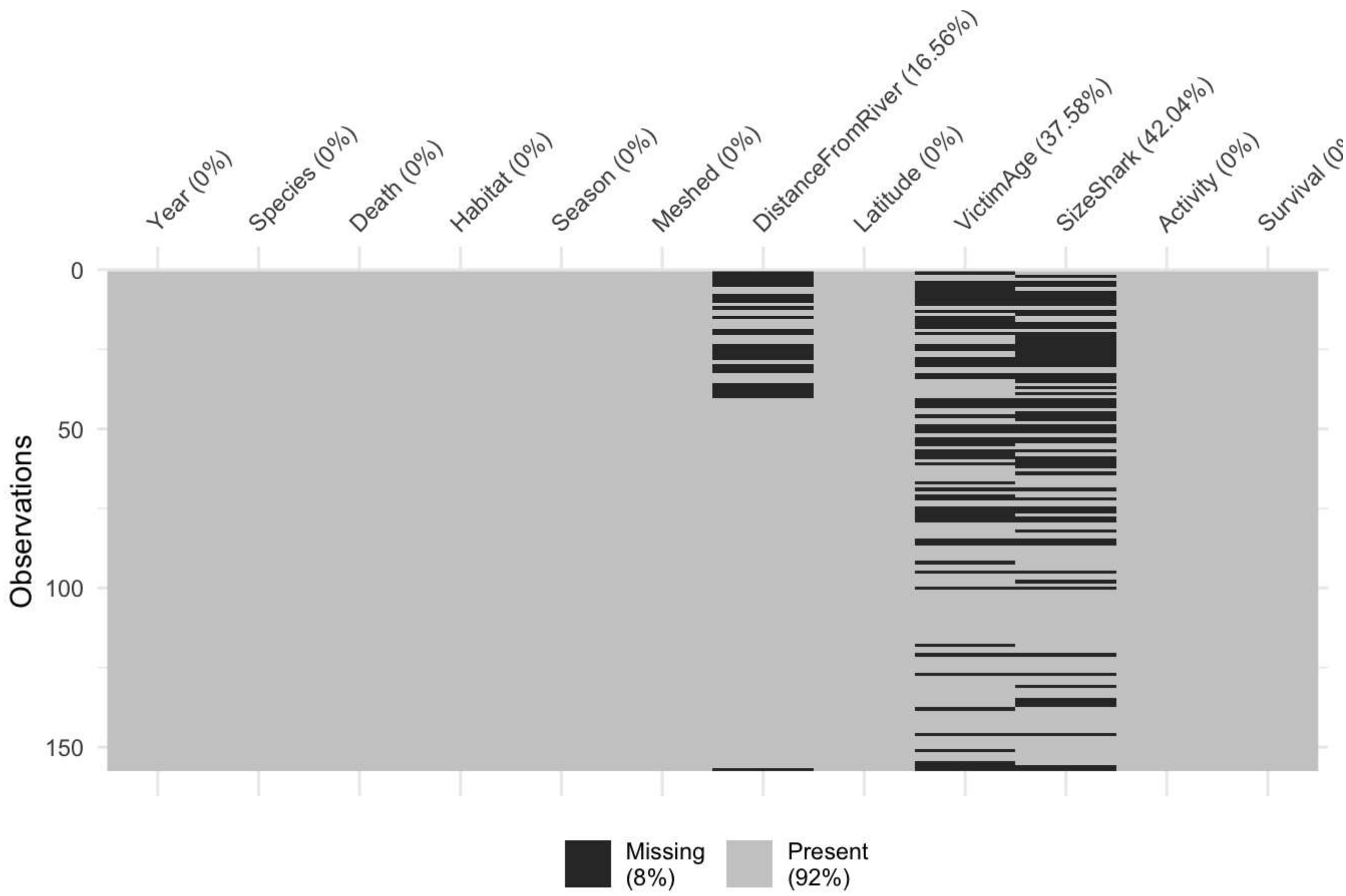

- No correlations > 0.6

- Missing data in 3 predictors. Approach used for missing data was listwise deletion, i.e.�only use complete cases for modelling

MODELLING

Use only complete cases

```
bull.cc <- na.omit(bull)
```

START MODEL: Survival ~ Year + Habitat + Season + Meshed + DistanceFromRiver + Latitude + Activity + VictimAge + SizeShark

```
m1 <- glm(Survival ~ Year + Habitat + Season + Meshed + DistanceFromRiver + Latitude + Activi
ty + VictimAge + SizeShark, data = bull.cc, family=binomial)

drop1(m1, test="Chisq")
```

```
## Single term deletions
##
## Model:
## Survival ~ Year + Habitat + Season + Meshed + DistanceFromRiver +
##     Latitude + Activity + VictimAge + SizeShark
##                   Df Deviance    AIC     LRT Pr(>Chi)
## <none>                 44.661 80.661
## Year               1   60.382 94.382 15.7212 7.34e-05 ***
## Habitat            8   62.634 82.634 17.9732  0.02143 *
## Season             2   50.082 82.082  5.4210  0.06651 .
## Meshed             1   48.725 82.725  4.0641  0.04381 *
## DistanceFromRiver  1   51.515 85.515  6.8548  0.00884 **
## Latitude           1   45.533 79.533  0.8721  0.35039
## Activity           1   45.698 79.698  1.0374  0.30843
## VictimAge          1   44.871 78.871  0.2099  0.64687
## SizeShark          1   48.055 82.055  3.3943  0.06542 .
## ---
## Signif. codes:  0 '***' 0.001 '**' 0.01 '*' 0.05 '.' 0.1 ' ' 1
```

Remove VictimAge

```
m2 <- update(m1, .~. - VictimAge)
drop1(m2, test="Chisq")
```

```
## Single term deletions
##
## Model:
## Survival ~ Year + Habitat + Season + Meshed + DistanceFromRiver +
##     Latitude + Activity + SizeShark
##                   Df Deviance    AIC     LRT  Pr(>Chi)
## <none>                 44.871 78.871
## Year               1   60.497 92.497 15.6261 7.718e-05 ***
## Habitat            8   62.909 80.909 18.0381  0.020943 *
## Season             2   51.194 81.194  6.3234  0.042353 *
## Meshed             1   49.602 81.602  4.7319  0.029608 *
## DistanceFromRiver  1   51.594 83.594  6.7239  0.009513 **
## Latitude           1   45.863 77.863  0.9920  0.319263
## Activity           1   46.176 78.176  1.3055  0.253214
## SizeShark          1   48.260 80.260  3.3898  0.065602 .
## ---
## Signif. codes:  0 '***' 0.001 '**' 0.01 '*' 0.05 '.' 0.1 ' ' 1
```

Remove Latitude

```
m3 <- update(m2, .~. - Latitude)
drop1(m3, test="Chisq")
```

```
## Single term deletions
##
## Model:
## Survival ~ Year + Habitat + Season + Meshed + DistanceFromRiver +
##     Activity + SizeShark
##                   Df Deviance    AIC     LRT  Pr(>Chi)
## <none>                 45.863 77.863
## Year               1   60.694 90.694 14.8317 0.0001175 ***
## Habitat            8   66.381 82.381 20.5184 0.0085429 **
## Season             2   51.263 79.263  5.4009 0.0671751 .
## Meshed             1   50.279 80.279  4.4163 0.0355975 *
## DistanceFromRiver  1   51.979 81.979  6.1168 0.0133900 *
## Activity           1   46.649 76.649  0.7864 0.3751763
## SizeShark          1   50.246 80.246  4.3830 0.0362985 *
## ---
## Signif. codes:  0 '***' 0.001 '**' 0.01 '*' 0.05 '.' 0.1 ' ' 1
```

Remove Activity

```
m4 <- update(m3, .~. - Activity)
drop1(m4, test="Chisq")
```

```
## Single term deletions
##
## Model:
## Survival ~ Year + Habitat + Season + Meshed + DistanceFromRiver +
##     SizeShark
##                   Df Deviance    AIC     LRT  Pr(>Chi)
## <none>                 46.649 76.649
## Year               1   61.833 89.833 15.1843 9.751e-05 ***
## Habitat            8   66.658 80.658 20.0094   0.01030 *
## Season             2   51.375 77.375  4.7259   0.09414 .
## Meshed             1   50.286 78.286  3.6371   0.05651 .
## DistanceFromRiver  1   52.206 80.206  5.5569   0.01841 *
## SizeShark          1   50.678 78.678  4.0290   0.04472 *
## ---
## Signif. codes:  0 '***' 0.001 '**' 0.01 '*' 0.05 '.' 0.1 ' ' 1
```

Remove Season

```
m5 <- update(m4, .~. - Season)
drop1(m5, test="Chisq")
```

```
## Single term deletions
##
## Model:
## Survival ~ Year + Habitat + Meshed + DistanceFromRiver + SizeShark
##                   Df Deviance    AIC     LRT  Pr(>Chi)
## <none>                 51.375 77.375
## Year               1   66.871 90.871 15.4958 8.269e-05 ***
## Habitat            8   69.174 79.174 17.7986   0.02279 *
## Meshed             1   53.573 77.573  2.1978   0.13821
## DistanceFromRiver  1   57.748 81.748  6.3728   0.01159 *
## SizeShark          1   53.607 77.607  2.2325   0.13513
## ---
## Signif. codes:  0 '***' 0.001 '**' 0.01 '*' 0.05 '.' 0.1 ' ' 1
```

Remove Meshed

```
m6 <- update(m5, .~. -Meshed)
drop1(m6, test="Chisq")
```

```
## Single term deletions
##
## Model:
## Survival ~ Year + Habitat + DistanceFromRiver + SizeShark
##                   Df Deviance    AIC     LRT  Pr(>Chi)
## <none>                 53.573 77.573
## Year               1   70.015 92.015 16.4423 5.015e-05 ***
## Habitat            8   70.317 78.317 16.7442   0.03289 *
## DistanceFromRiver  1   58.737 80.737  5.1645   0.02305 *
## SizeShark          1   55.028 77.028  1.4554   0.22767
## ---
## Signif. codes:  0 '***' 0.001 '**' 0.01 '*' 0.05 '.' 0.1 ' ' 1
```

Remove SizeShark

```
m7 <- update(m6, .~. -SizeShark)
drop1(m7, test="Chisq")
```

```
## Single term deletions
##
## Model:
## Survival ~ Year + Habitat + DistanceFromRiver
##                   Df Deviance    AIC     LRT  Pr(>Chi)
## <none>                 55.028 77.028
## Year               1   72.370 92.370 17.3422 3.122e-05 ***
## Habitat            8   70.510 76.510 15.4821   0.05042 .
## DistanceFromRiver  1   60.490 80.490  5.4624   0.01943 *
## ---
## Signif. codes:  0 '***' 0.001 '**' 0.01 '*' 0.05 '.' 0.1 ' ' 1
```

Remove Habitat

```
m8 <-update(m7, .~. -Habitat)
drop1(m8, test="Chisq")
```

```
## Single term deletions
##
## Model:
## Survival ~ Year + DistanceFromRiver
##                   Df Deviance    AIC     LRT  Pr(>Chi)
## <none>                 70.510 76.510
## Year               1   88.575 92.575 18.0649 2.135e-05 ***
## DistanceFromRiver  1   72.347 76.347  1.8373    0.1753
## ---
## Signif. codes:  0 '***' 0.001 '**' 0.01 '*' 0.05 '.' 0.1 ' ' 1
```

Remove DistanceFromRiver

```
m9 <- update(m8, .~. -DistanceFromRiver)
drop1(m9, test="Chisq")
```

```
## Single term deletions
##
## Model:
## Survival ~ Year
##        Df Deviance    AIC    LRT  Pr(>Chi)
## <none>      72.347 76.347
## Year    1   95.524 97.524 23.176 1.478e-06 ***
## ---
## Signif. codes:  0 '***' 0.001 '**' 0.01 '*' 0.05 '.' 0.1 ' ' 1
```

Final model: Survival ~ Year

```
summary(m9)
```

```
##
## Call:
## glm(formula = Survival ~ Year, family = binomial, data = bull.cc)
##
## Deviance Residuals:
##     Min       1Q   Median       3Q      Max
## -2.0748  -0.8357   0.4433   0.6932   1.7084
##
## Coefficients:
##               Estimate Std. Error z value Pr(>|z|)
## (Intercept) -78.883333  19.269142  -4.094 4.24e-05 ***
## Year          0.040295   0.009841   4.094 4.23e-05 ***
## ---
## Signif. codes:  0 '***' 0.001 '**' 0.01 '*' 0.05 '.' 0.1 ' ' 1
##
## (Dispersion parameter for binomial family taken to be 1)
##
##     Null deviance: 95.524  on 68  degrees of freedom
## Residual deviance: 72.347  on 67  degrees of freedom
## AIC: 76.347
##
## Number of Fisher Scoring iterations: 4
```

Diagnostics

```
res <- simulateResiduals(fittedModel = m9, n = 250)
plot(res)
```

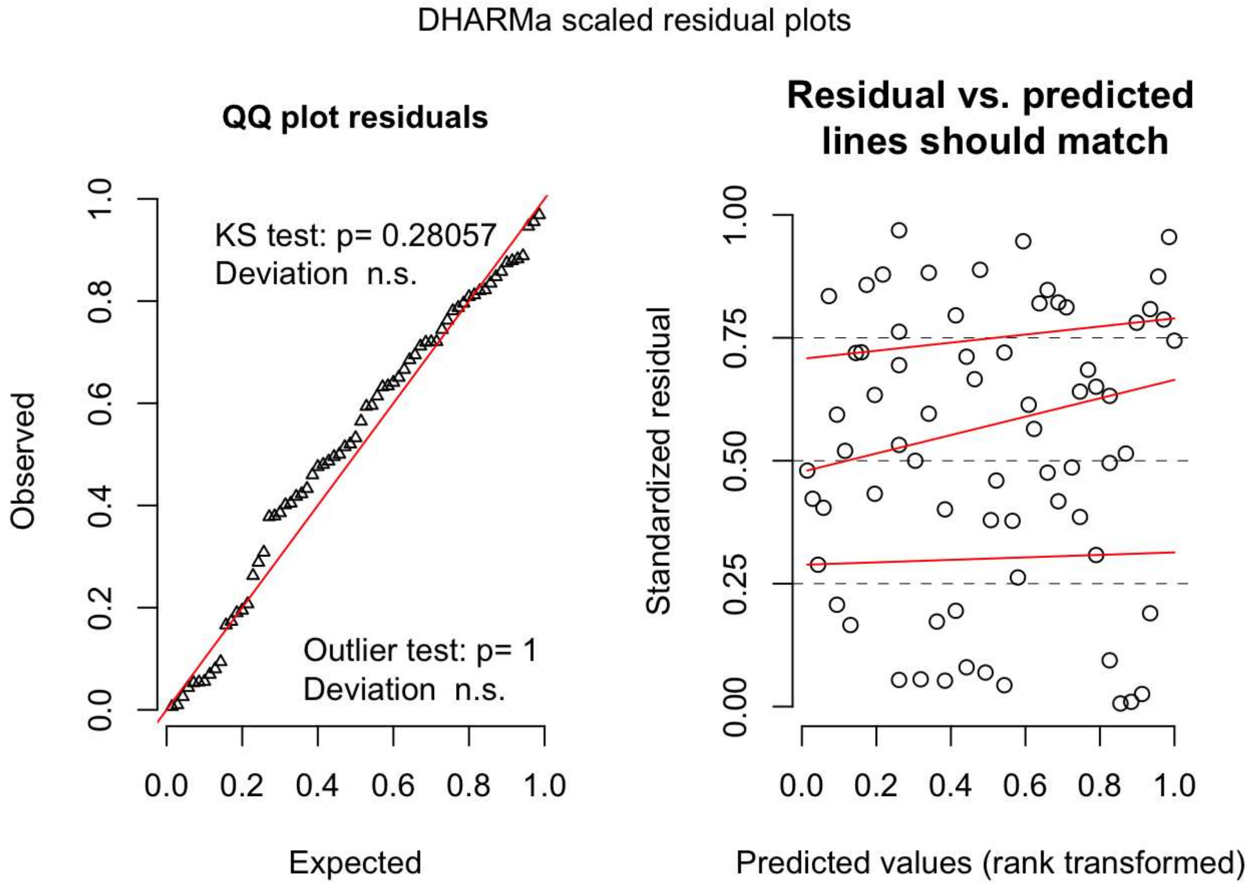

Test for temporal autocorrelation

```
resYear <- recalculateResiduals(res, group=bull.cc$Year, aggregateBy = mean)
testTemporalAutocorrelation(resYear, time=unique(bull.cc$Year))
```

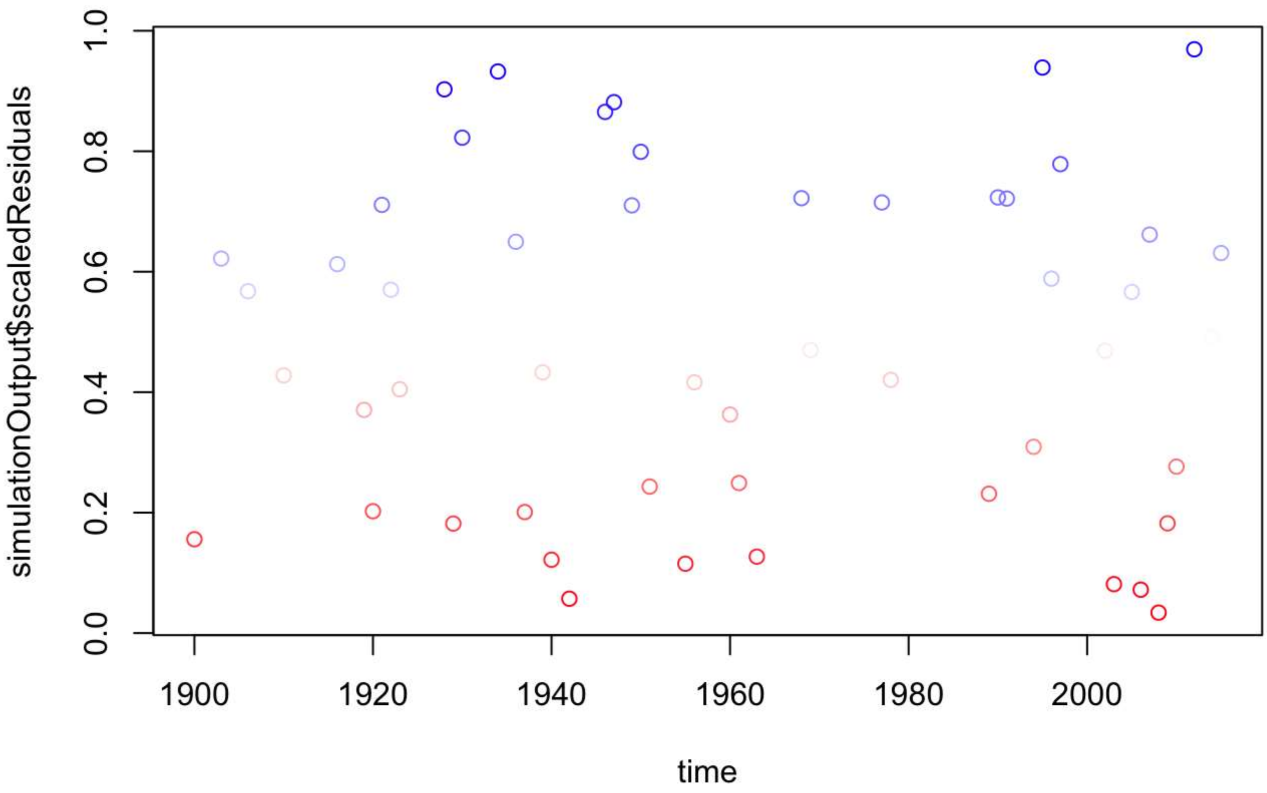


```
##
##  Durbin-Watson test
##
## data:  simulationOutput$scaledResiduals ~ 1
## DW = 1.9223, p-value = 0.7801
## alternative hypothesis: true autocorrelation is not 0
```

Test for overdispersion

```
testDispersion(res)
```

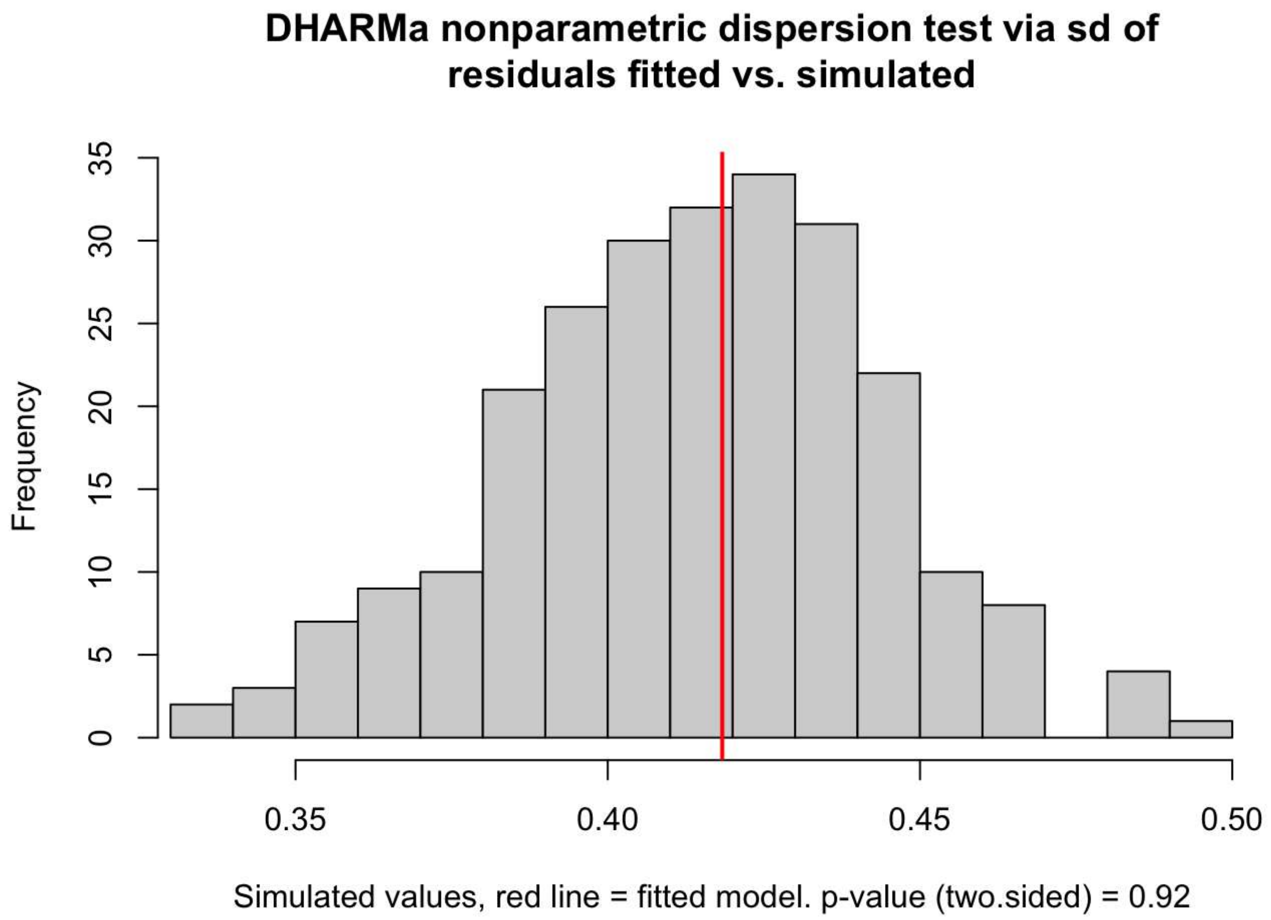


```
##
##  DHARMa nonparametric dispersion test via sd of residuals fitted vs.
##  simulated
##
## data:  simulationOutput
## ratioObsSim = 1.0108, p-value = 0.92
## alternative hypothesis: two.sided
```

Test for zero inflation

```
testZeroInflation(res)
```

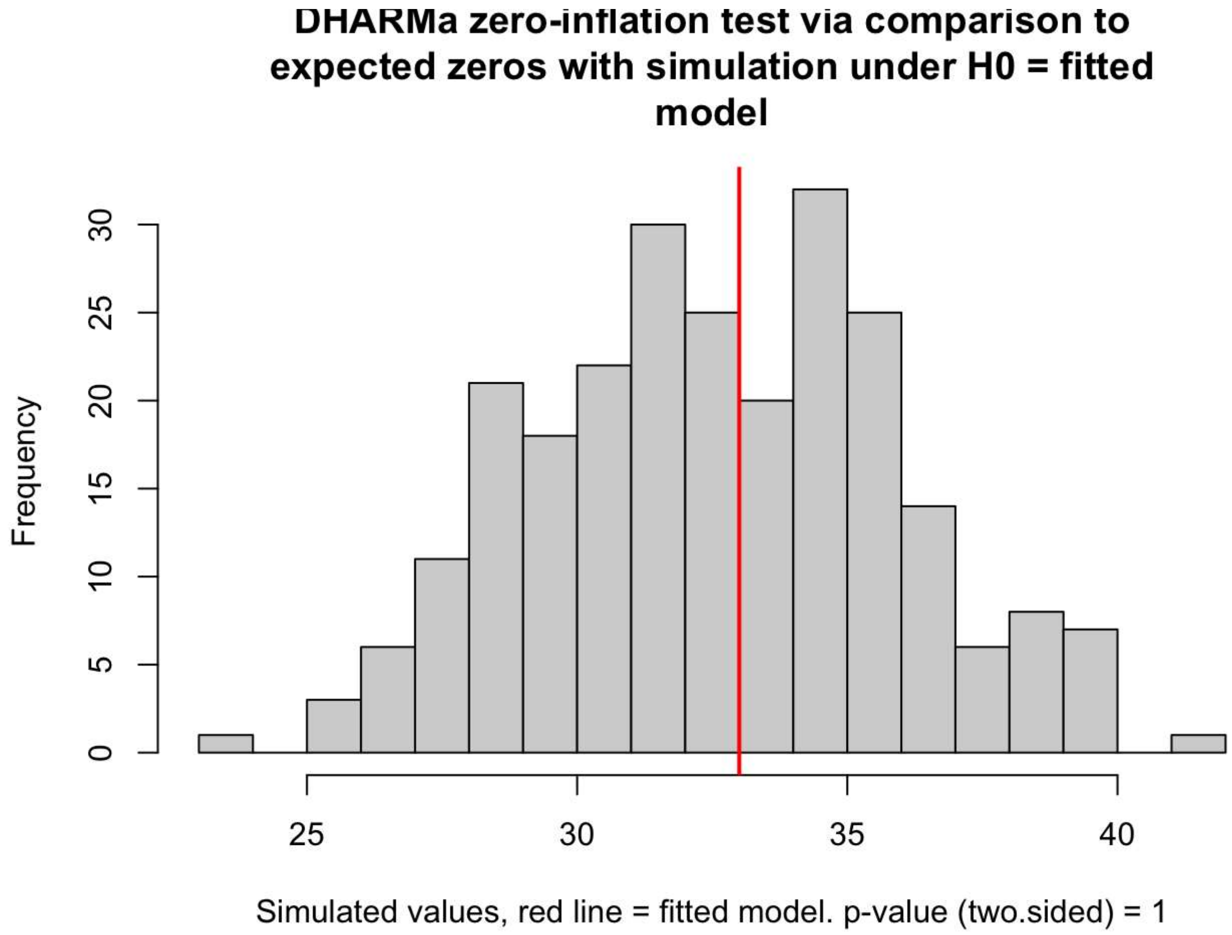


```
##
##  DHARMa zero-inflation test via comparison to expected zeros with
##  simulation under H0 = fitted model
##
## data:  simulationOutput
## ratioObsSim = 0.99794, p-value = 1
## alternative hypothesis: two.sided
```

Plot results

Predict means and standard errors from model

```
nd <- data.frame(Year = seq(from=min(bull.cc$Year), to=max(bull.cc$Year), length=100))
pred <- predict(m9, nd, se.fit=T)
nd$Survival <- inv.logit(pred$fit)

nd$SE_upper <- inv.logit(pred$fit + pred$se.fit)
nd$SE_lower <- inv.logit(pred$fit - pred$se.fit)
print(nd)
```

```
##        Year   Survival  SE_upper   SE_lower
## 1  1900.000 0.08923715 0.1564012 0.04923240
## 2  1901.162 0.09311521 0.1612966 0.05196880
## 3  1902.323 0.09714383 0.1663212 0.05484623
## 4  1903.485 0.10132726 0.1714769 0.05787072
## 5  1904.646 0.10566978 0.1767657 0.06104843
## 6  1905.808 0.11017557 0.1821893 0.06438560
## 7  1906.970 0.11484883 0.1877497 0.06788859
## 8  1908.131 0.11969364 0.1934484 0.07156382
## 9  1909.293 0.12471403 0.1992873 0.07541774
## 10 1910.455 0.12991392 0.2052678 0.07945682
## 11 1911.616 0.13529711 0.2113915 0.08368755
## 12 1912.778 0.14086724 0.2176597 0.08811635
## 13 1913.939 0.14662781 0.2240739 0.09274957
## 14 1915.101 0.15258210 0.2306353 0.09759344
## 15 1916.263 0.15873322 0.2373450 0.10265406
## 16 1917.424 0.16508400 0.2442040 0.10793730
## 17 1918.586 0.17163704 0.2512134 0.11344878
## 18 1919.747 0.17839461 0.2583739 0.11919381
## 19 1920.909 0.18535871 0.2656862 0.12517736
## 20 1922.071 0.19253096 0.2731511 0.13140395
## 21 1923.232 0.19991263 0.2807690 0.13787764
## 22 1924.394 0.20750459 0.2885403 0.14460192
## 23 1925.556 0.21530727 0.2964652 0.15157969
## 24 1926.717 0.22332068 0.3045440 0.15881314
## 25 1927.879 0.23154432 0.3127767 0.16630373
## 26 1929.040 0.23997723 0.3211631 0.17405209
## 27 1930.202 0.24861790 0.3297031 0.18205798
## 28 1931.364 0.25746430 0.3383964 0.19032020
## 29 1932.525 0.26651382 0.3472422 0.19883654
## 30 1933.687 0.27576329 0.3562402 0.20760376
## 31 1934.848 0.28520895 0.3653893 0.21661749
## 32 1936.010 0.29484643 0.3746886 0.22587221
## 33 1937.172 0.30467076 0.3841369 0.23536125
## 34 1938.333 0.31467636 0.3937328 0.24507674
## 35 1939.495 0.32485703 0.4034745 0.25500964
## 36 1940.657 0.33520598 0.4133601 0.26514972
## 37 1941.818 0.34571579 0.4233873 0.27548561
## 38 1942.980 0.35637847 0.4335534 0.28600488
## 39 1944.141 0.36718545 0.4438552 0.29669403
## 40 1945.303 0.37812762 0.4542893 0.30753868
## 41 1946.465 0.38919532 0.4648514 0.31852357
## 42 1947.626 0.40037840 0.4755368 0.32963279
## 43 1948.788 0.41166625 0.4863400 0.34084985
## 44 1949.949 0.42304781 0.4972548 0.35215786
## 45 1951.111 0.43451164 0.5082741 0.36353969
## 46 1952.273 0.44604595 0.5193901 0.37497817
## 47 1953.434 0.45763866 0.5305937 0.38645621
## 48 1954.596 0.46927743 0.5418752 0.39795704
## 49 1955.758 0.48094971 0.5532237 0.40946430
## 50 1956.919 0.49264282 0.5646271 0.42096226
## 51 1958.081 0.50434399 0.5760728 0.43243589
## 52 1959.242 0.51604040 0.5875469 0.44387100
## 53 1960.404 0.52771926 0.5990347 0.45525431
## 54 1961.566 0.53936787 0.6105208 0.46657355
## 55 1962.727 0.55097363 0.6219890 0.47781740
## 56 1963.889 0.56252415 0.6334230 0.48897561
## 57 1965.051 0.57400727 0.6448057 0.50003891
```

```
## 58  1966.212 0.58541112 0.6561200 0.51099901
## 59  1967.374 0.59672418 0.6673489 0.52184852
## 60  1968.535 0.60793527 0.6784754 0.53258094
## 61  1969.697 0.61903368 0.6894830 0.54319055
## 62  1970.859 0.63000912 0.7003557 0.55367236
## 63  1972.020 0.64085181 0.7110779 0.56402204
## 64  1973.182 0.65155250 0.7216350 0.57423583
## 65  1974.343 0.66210246 0.7320133 0.58431049
## 66  1975.505 0.67249357 0.7422000 0.59424324
## 67  1976.667 0.68271825 0.7521835 0.60403168
## 68  1977.828 0.69276956 0.7619531 0.61367378
## 69  1978.990 0.70264113 0.7714994 0.62316780
## 70  1980.152 0.71232723 0.7808143 0.63251227
## 71  1981.313 0.72182273 0.7898906 0.64170596
## 72  1982.475 0.73112311 0.7987224 0.65074784
## 73  1983.636 0.74022445 0.8073051 0.65963708
## 74  1984.798 0.74912343 0.8156350 0.66837302
## 75  1985.960 0.75781730 0.8237097 0.67695515
## 76  1987.121 0.76630388 0.8315275 0.68538313
## 77  1988.283 0.77458153 0.8390880 0.69365672
## 78  1989.444 0.78264914 0.8463915 0.70177585
## 79  1990.606 0.79050612 0.8534391 0.70974055
## 80  1991.768 0.79815232 0.8602329 0.71755100
## 81  1992.929 0.80558807 0.8667756 0.72520747
## 82  1994.091 0.81281415 0.8730704 0.73271037
## 83  1995.253 0.81983171 0.8791212 0.74006021
## 84  1996.414 0.82664230 0.8849323 0.74725762
## 85  1997.576 0.83324780 0.8905087 0.75430334
## 86  1998.737 0.83965044 0.8958554 0.76119820
## 87  1999.899 0.84585271 0.9009781 0.76794315
## 88  2001.061 0.85185741 0.9058825 0.77453925
## 89  2002.222 0.85766756 0.9105746 0.78098763
## 90  2003.384 0.86328641 0.9150606 0.78728954
## 91  2004.545 0.86871740 0.9193469 0.79344632
## 92  2005.707 0.87396414 0.9234397 0.79945938
## 93  2006.869 0.87903038 0.9273456 0.80533022
## 94  2008.030 0.88392004 0.9310710 0.81106044
## 95  2009.192 0.88863708 0.9346225 0.81665168
## 96  2010.354 0.89318561 0.9380063 0.82210568
## 97  2011.515 0.89756977 0.9412289 0.82742424
## 98  2012.677 0.90179377 0.9442966 0.83260921
## 99  2013.838 0.90586185 0.9472155 0.83766250
## 100 2015.000 0.90977828 0.9499917 0.84258609
```

Plots model predictions

```
ggplot(nd, aes(x=Year, y= Survival)) + geom_line() +
geom_ribbon(aes(ymax=SE_upper, ymin=SE_lower), alpha=0.2) +
geom_jitter(data=bull.cc, width=0.02, height=0.02) +
ylab("Probability of Survival") +
xlab("Year") +
ggtitle("Bull Sharks")
```

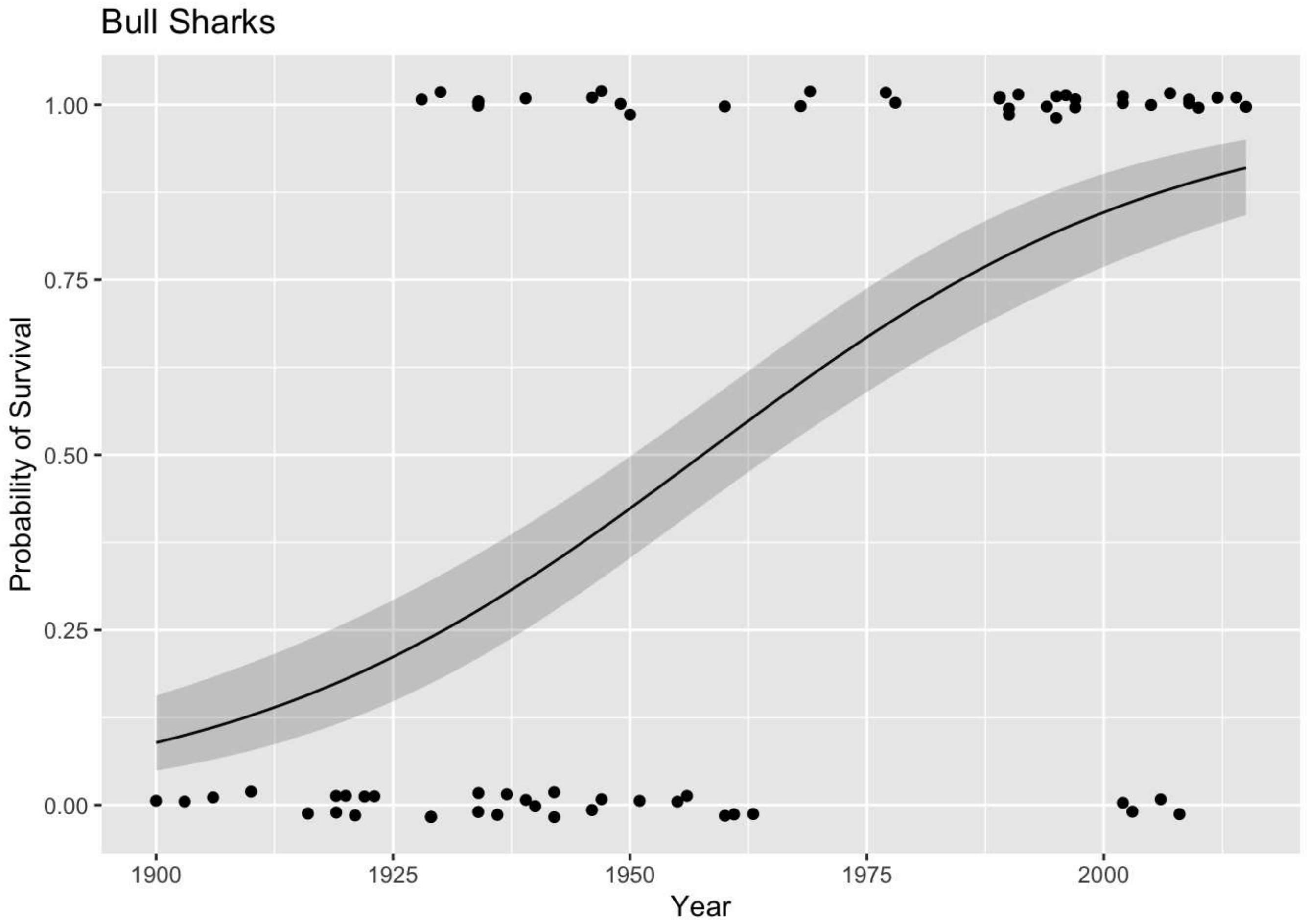

Plot Activity for comparison with other spp. Note that Activity is (just) significant when tested by itself.

```
ma <- glm(Survival ~  Activity, data = bull.cc, family=binomial)
drop1(ma, test = "Chisq")
```

```
## Single term deletions
##
## Model:
## Survival ~ Activity
##          Df Deviance    AIC   LRT Pr(>Chi)
## <none>        91.647 95.647
## Activity  1   95.524 97.524 3.877  0.04895 *
## ---
## Signif. codes:  0 '***' 0.001 '**' 0.01 '*' 0.05 '.' 0.1 ' ' 1
```

```
nd <- data.frame(Activity = levels(bull.cc$Activity))
pred <- predict(ma, nd, se.fit=T)
nd$Survival <- inv.logit(pred$fit)

nd$SE_upper <- inv.logit(pred$fit + pred$se.fit)
nd$SE_lower <- inv.logit(pred$fit - pred$se.fit)
nd$Activity <- factor(nd$Activity, labels=c("In-water","On-water"))
print(nd)
```

```
##   Activity  Survival  SE_upper  SE_lower
## 1 In-water 0.4745763 0.5396497 0.4103551
## 2 On-water 0.8000000 0.8981525 0.6446777
```

```
ggplot(nd, aes(x=Activity, y= Survival)) + geom_bar(stat="identity") +
geom_linerange(aes(ymax=SE_upper, ymin=SE_lower)) +
ylab("Probability of Survival") +
xlab("Victim activity") +
ggtitle("Bull Sharks")
```

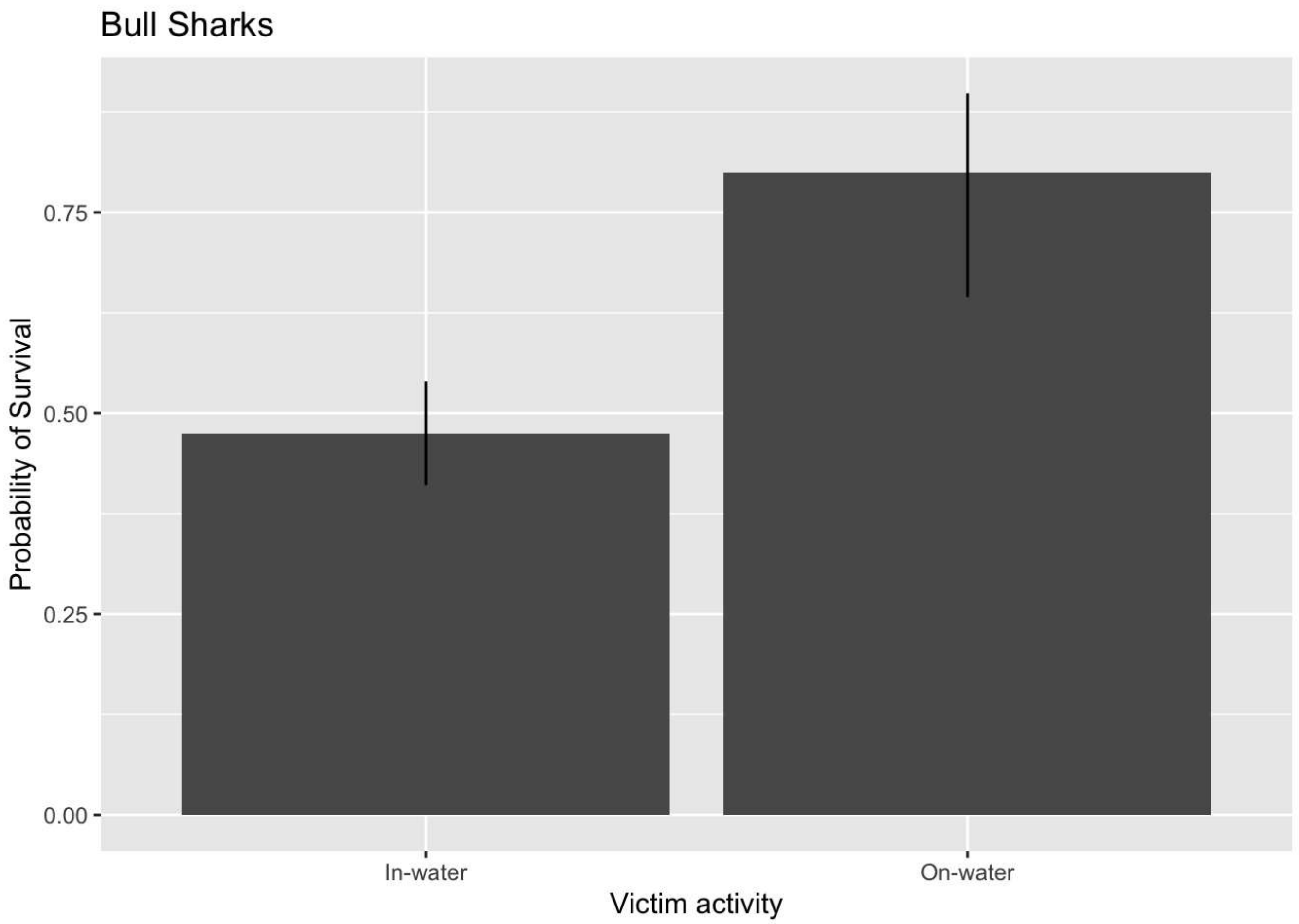

Supplement: Supplementary file 1 — Supplementary Information 1. [file 41598_2022_16950_MOESM1_ESM.pdf]
