# Supplementary material for: Increased shark bite survivability revealed by two centuries of Australian records
Source: Sci Rep. 2022 Aug 19;12:14121. doi: 10.1038/s41598-022-16950-5 (PMC9391475; doi:10.1038/s41598-022-16950-5)

# TIGER SHARKS

- Load data and libraries

```
dd <- read.csv("SharkDeaths.csv")
library(DHARMa)
library(ggplot2)
library(GGally)
library(boot)
library(visdat)
```

- remove TimeOfDay
- subset for the species
- add Survival

```
dd$TimeOfDay <- NULL
dd$Survival <- 1 - dd$Death
tiger <- dd[dd$Species=="TS",]
```

- inspect missing data and correlations between predictor variables

```
vis_miss(tiger)
```

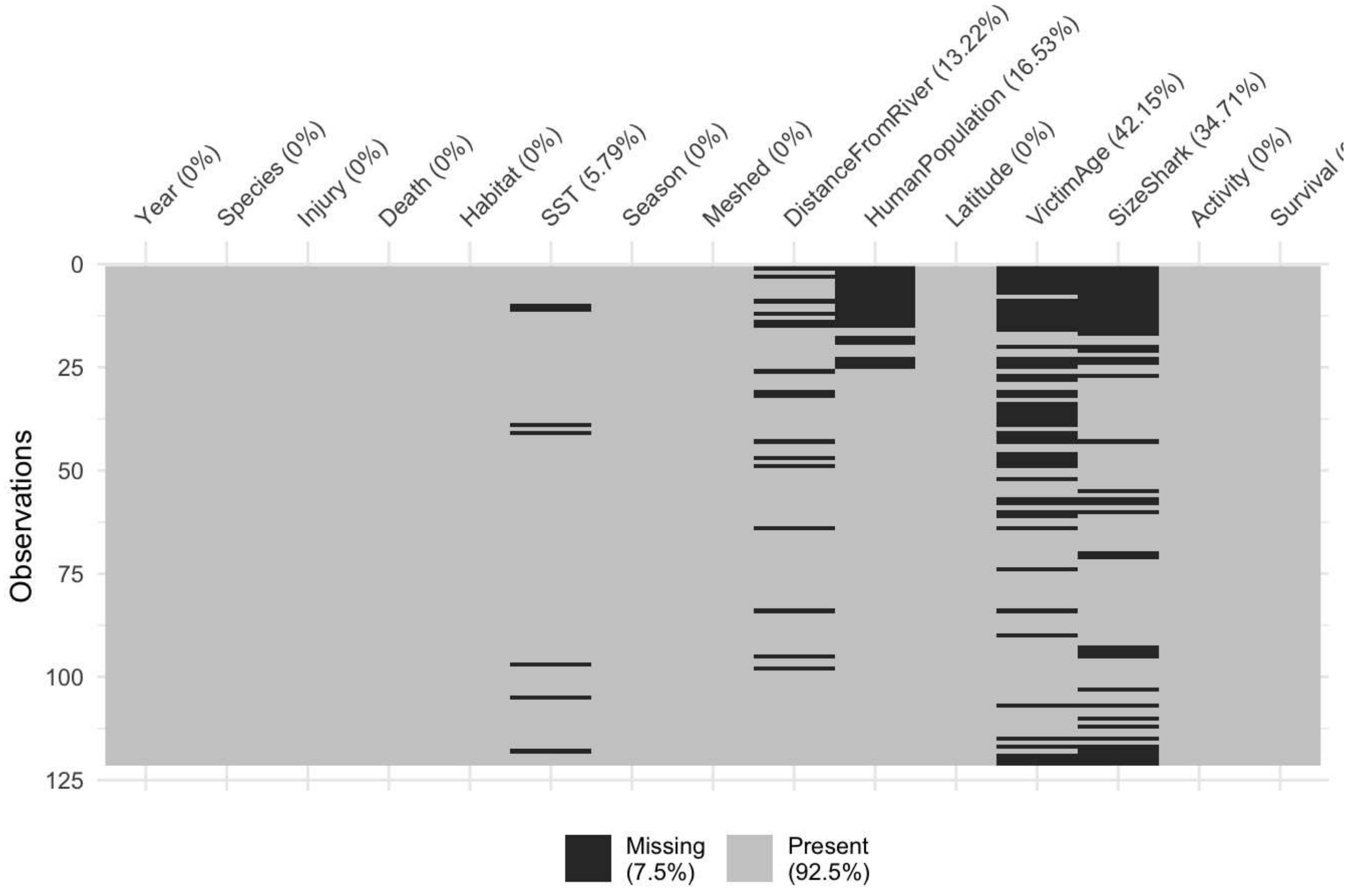


```
numericPredictors <- names(tiger)[names(tiger) %in%
                        c("Habitat","Species","Season","Activity") == F]
ggpairs(tiger[,numericPredictors])
```

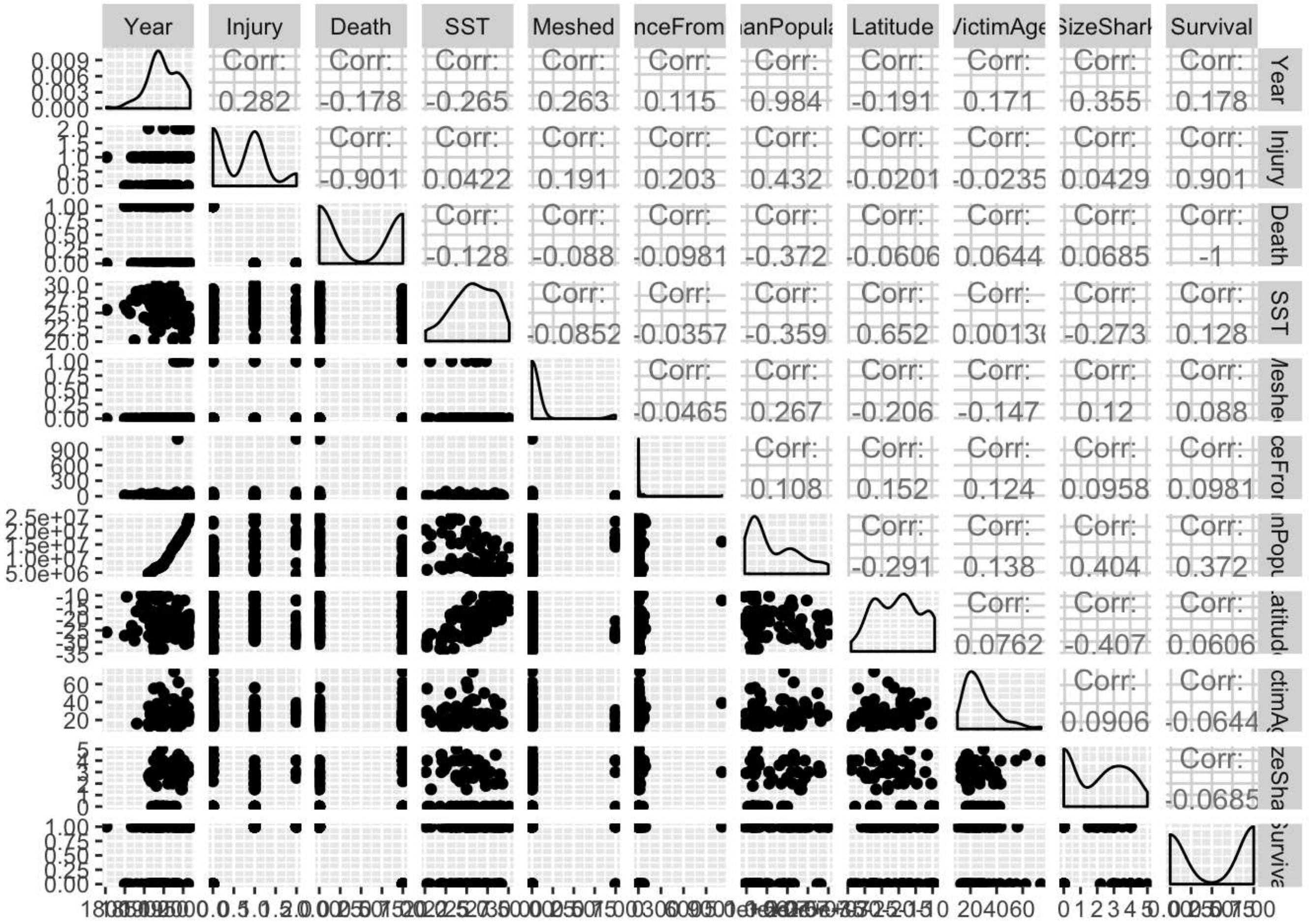

- Remove Human Population (correlated with Year)
- Remove Injury
- SST and latitude are correlated (>0.6). Remove SST.

```
tiger$ HumanPopulation <- NULL
tiger$ Injury <- NULL
tiger$ TimeOfDay <- NULL
tiger$ SST <- NULL

numericPredictors <- names(tiger)[names(tiger) %in%
                        c("Habitat","Species","Season","Activity") == F]
ggpairs(tiger[,numericPredictors])
```

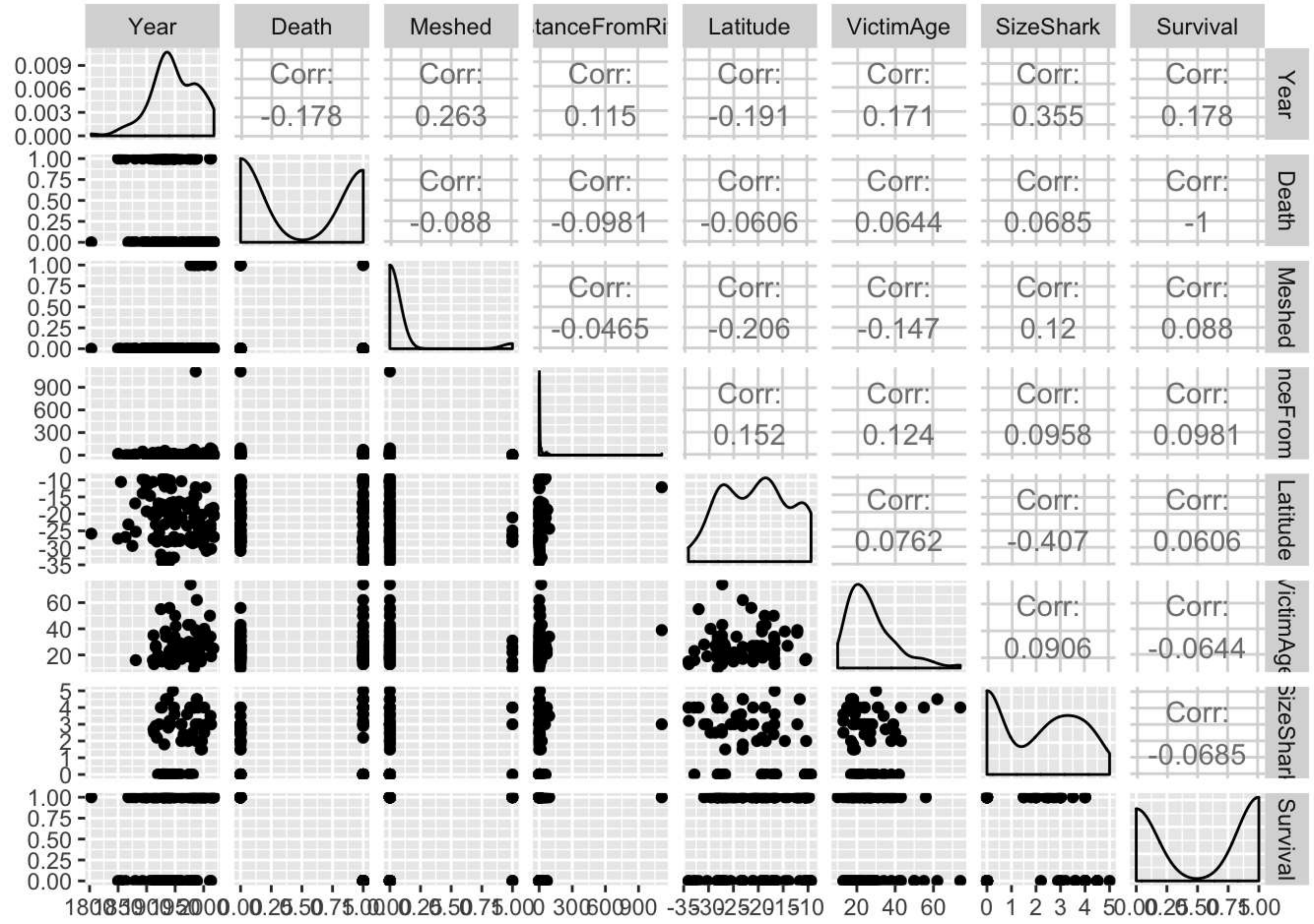


```
vis_miss(tiger)
```

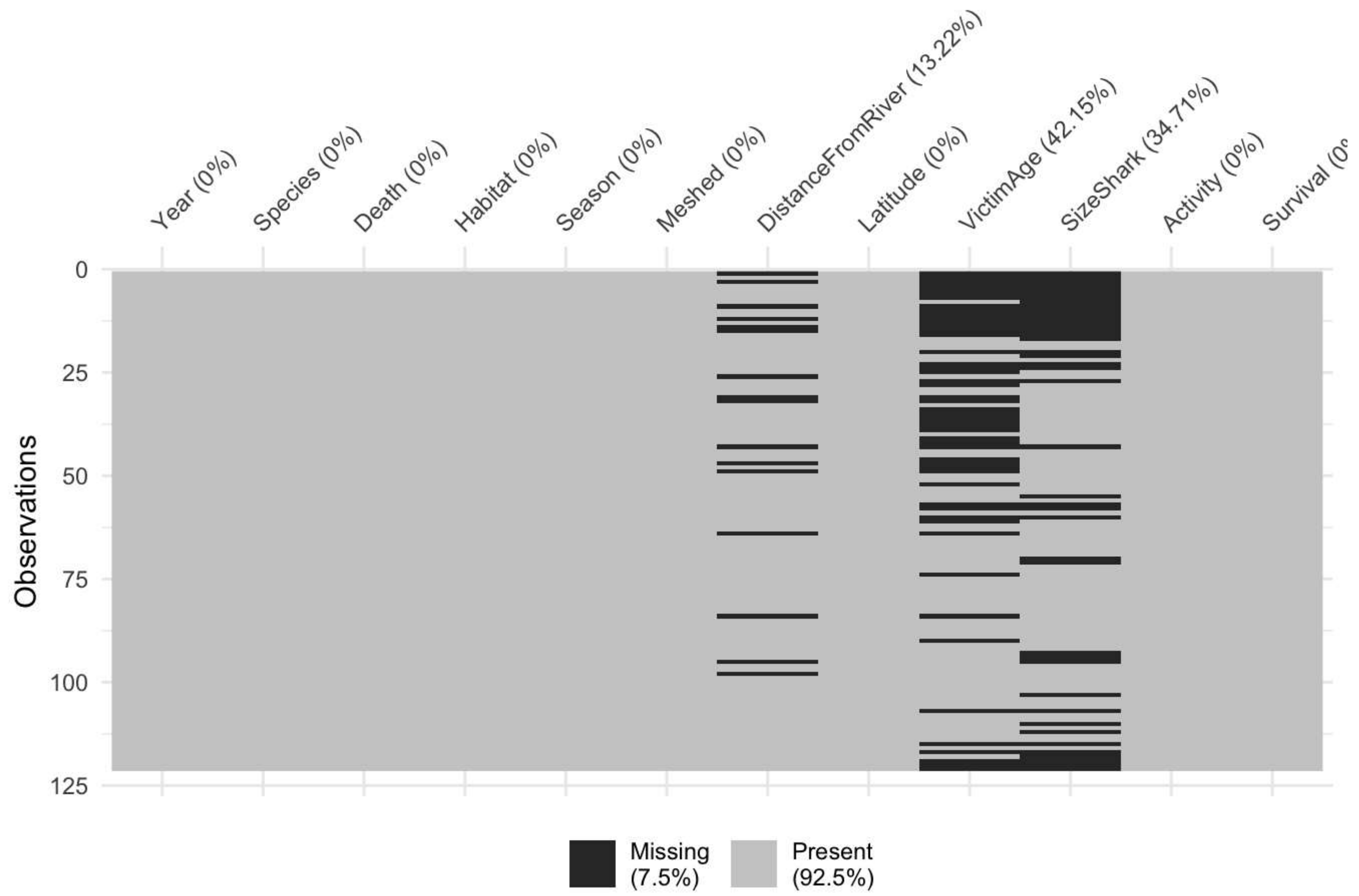


- No correlations > 0.6

- Missing data in 3 predictors. Approach used for missing data was listwise deletion, i.e.�only use complete cases for modelling

# MODELLING

Use only complete cases

```
tiger.cc <- na.omit(tiger)
```

START MODEL: Survival ~ Year + Habitat + Season + Meshed + DistanceFromRiver + Latitude + Activity + VictimAge + SizeShark

```
m1 <- glm(Survival ~ Year + Habitat + Season + Meshed + DistanceFromRiver + Latitude + Activi
ty + VictimAge + SizeShark, data = tiger.cc, family=binomial)

drop1(m1, test="Chisq")
```

```
## Single term deletions
##
## Model:
## Survival ~ Year + Habitat + Season + Meshed + DistanceFromRiver +
##     Latitude + Activity + VictimAge + SizeShark
##                   Df Deviance    AIC     LRT  Pr(>Chi)
## <none>                 45.838 79.838
## Year               1   46.116 78.116  0.2771 0.5986038
## Habitat            6   53.329 75.329  7.4906 0.2778440
## Season             3   46.813 74.813  0.9746 0.8073979
## Meshed             1   45.856 77.856  0.0172 0.8955227
## DistanceFromRiver  1   47.163 79.163  1.3249 0.2497184
## Latitude           1   48.868 80.868  3.0293 0.0817714 .
## Activity           1   60.243 92.243 14.4049 0.0001474 ***
## VictimAge          1   45.951 77.951  0.1126 0.7372252
## SizeShark          1   46.820 78.820  0.9815 0.3218294
## ---
## Signif. codes:  0 '***' 0.001 '**' 0.01 '*' 0.05 '.' 0.1 ' ' 1
```

Remove Season

```
m2 <- update(m1, .~. - Season)
drop1(m2, test="Chisq")
```

```
## Single term deletions
##
## Model:
## Survival ~ Year + Habitat + Meshed + DistanceFromRiver + Latitude +
##     Activity + VictimAge + SizeShark
##                   Df Deviance    AIC     LRT  Pr(>Chi)
## <none>                 46.813 74.813
## Year               1   47.046 73.046  0.2327 0.6295306
## Habitat            6   55.193 71.193  8.3800 0.2115668
## Meshed             1   46.904 72.904  0.0914 0.7624046
## DistanceFromRiver  1   47.979 73.979  1.1659 0.2802498
## Latitude           1   49.067 75.067  2.2539 0.1332745
## Activity           1   60.462 86.462 13.6488 0.0002204 ***
## VictimAge          1   47.214 73.214  0.4015 0.5263220
## SizeShark          1   47.600 73.600  0.7872 0.3749420
## ---
## Signif. codes:  0 '***' 0.001 '**' 0.01 '*' 0.05 '.' 0.1 ' ' 1
```

Remove Habitat

```
m3 <- update(m2, .~. - Habitat)
drop1(m3, test="Chisq")
```

```
## Single term deletions
##
## Model:
## Survival ~ Year + Meshed + DistanceFromRiver + Latitude + Activity +
##     VictimAge + SizeShark
##                   Df Deviance    AIC     LRT  Pr(>Chi)
## <none>                 55.193 71.193
## Year               1   55.281 69.281  0.0876 0.7672816
## Meshed             1   55.442 69.442  0.2492 0.6176709
## DistanceFromRiver  1   55.236 69.236  0.0428 0.8360805
## Latitude           1   57.956 71.956  2.7630 0.0964645 .
## Activity           1   69.010 83.010 13.8168 0.0002015 ***
## VictimAge          1   55.264 69.264  0.0710 0.7898456
## SizeShark          1   55.869 69.869  0.6758 0.4110537
## ---
## Signif. codes:  0 '***' 0.001 '**' 0.01 '*' 0.05 '.' 0.1 ' ' 1
```

Remove VictimAge

```
m4 <- update(m3, .~. - VictimAge)
drop1(m4, test="Chisq")
```

```
## Single term deletions
##
## Model:
## Survival ~ Year + Meshed + DistanceFromRiver + Latitude + Activity +
##     SizeShark
##                   Df Deviance    AIC     LRT  Pr(>Chi)
## <none>                 55.264 69.264
## Year               1   55.379 67.379  0.1149 0.7346160
## Meshed             1   55.489 67.489  0.2248 0.6354172
## DistanceFromRiver  1   55.305 67.305  0.0407 0.8400961
## Latitude           1   58.129 70.129  2.8651 0.0905177 .
## Activity           1   69.320 81.320 14.0562 0.0001774 ***
## SizeShark          1   55.935 67.935  0.6705 0.4128641
## ---
## Signif. codes:  0 '***' 0.001 '**' 0.01 '*' 0.05 '.' 0.1 ' ' 1
```

Remove Meshed

```
m5 <- update(m4, .~. - Meshed)
drop1(m5, test="Chisq")
```

```
## Single term deletions
##
## Model:
## Survival ~ Year + DistanceFromRiver + Latitude + Activity + SizeShark
##                   Df Deviance    AIC     LRT  Pr(>Chi)
## <none>                 55.489 67.489
## Year               1   55.686 65.686  0.1969 0.6571979
## DistanceFromRiver  1   55.569 65.569  0.0805 0.7766206
## Latitude           1   58.418 68.418  2.9288 0.0870110 .
## Activity           1   69.328 79.328 13.8396 0.0001991 ***
## SizeShark          1   56.182 66.182  0.6937 0.4048995
## ---
## Signif. codes:  0 '***' 0.001 '**' 0.01 '*' 0.05 '.' 0.1 ' ' 1
```

Remove DistanceFromRiver

```
m6 <- update(m5, .~. -DistanceFromRiver)
drop1(m6, test="Chisq")
```

```
## Single term deletions
##
## Model:
## Survival ~ Year + Latitude + Activity + SizeShark
##           Df Deviance    AIC     LRT  Pr(>Chi)
## <none>         55.569 65.569
## Year       1   55.719 63.719  0.1497 0.6988460
## Latitude   1   58.683 66.683  3.1141 0.0776173 .
## Activity   1   69.941 77.941 14.3714 0.0001501 ***
## SizeShark  1   56.208 64.208  0.6387 0.4241866
## ---
## Signif. codes:  0 '***' 0.001 '**' 0.01 '*' 0.05 '.' 0.1 ' ' 1
```

Remove SizeShark

```
m7 <- update(m6, .~. -SizeShark)
drop1(m7, test="Chisq")
```

```
## Single term deletions
##
## Model:
## Survival ~ Year + Latitude + Activity
##          Df Deviance    AIC     LRT  Pr(>Chi)
## <none>        56.208 64.208
## Year      1   56.540 62.540  0.3323 0.5642795
## Latitude  1   59.536 65.536  3.3280 0.0681084 .
## Activity  1   70.282 76.282 14.0737 0.0001758 ***
## ---
## Signif. codes:  0 '***' 0.001 '**' 0.01 '*' 0.05 '.' 0.1 ' ' 1
```

Remove Year

```
m8 <-update(m7, .~. -Year)
drop1(m8, test="Chisq")
```

```
## Single term deletions
##
## Model:
## Survival ~ Latitude + Activity
##          Df Deviance    AIC     LRT  Pr(>Chi)
## <none>        56.540 62.540
## Latitude  1   59.587 63.587  3.0464   0.08091 .
## Activity  1   74.506 78.506 17.9658 2.249e-05 ***
## ---
## Signif. codes:  0 '***' 0.001 '**' 0.01 '*' 0.05 '.' 0.1 ' ' 1
```

Remove Latitude

```
m9 <- update(m8, .~. -Latitude)
drop1(m9, test="Chisq")
```

```
## Single term deletions
##
## Model:
## Survival ~ Activity
##          Df Deviance    AIC    LRT  Pr(>Chi)
## <none>        59.587 63.587
## Activity  1   75.353 77.353 15.766 7.167e-05 ***
## ---
## Signif. codes:  0 '***' 0.001 '**' 0.01 '*' 0.05 '.' 0.1 ' ' 1
```

Final model: Survival ~ Activity

```
summary(m9)
```

```
##
## Call:
## glm(formula = Survival ~ Activity, family = binomial, data = tiger.cc)
##
## Deviance Residuals:
##     Min       1Q   Median       3Q      Max
## -2.2293  -0.8485  -0.8485   0.4172   1.5468
##
## Coefficients:
##             Estimate Std. Error z value Pr(>|z|)
## (Intercept)  -0.8362     0.3320  -2.518  0.01179 *
## ActivityON    3.2341     1.0960   2.951  0.00317 **
## ---
## Signif. codes:  0 '***' 0.001 '**' 0.01 '*' 0.05 '.' 0.1 ' ' 1
##
## (Dispersion parameter for binomial family taken to be 1)
##
##     Null deviance: 75.353  on 54  degrees of freedom
## Residual deviance: 59.587  on 53  degrees of freedom
## AIC: 63.587
##
## Number of Fisher Scoring iterations: 5
```

# Diagnostics

```
res <- simulateResiduals(fittedModel = m9, n = 250)
plot(res)
```

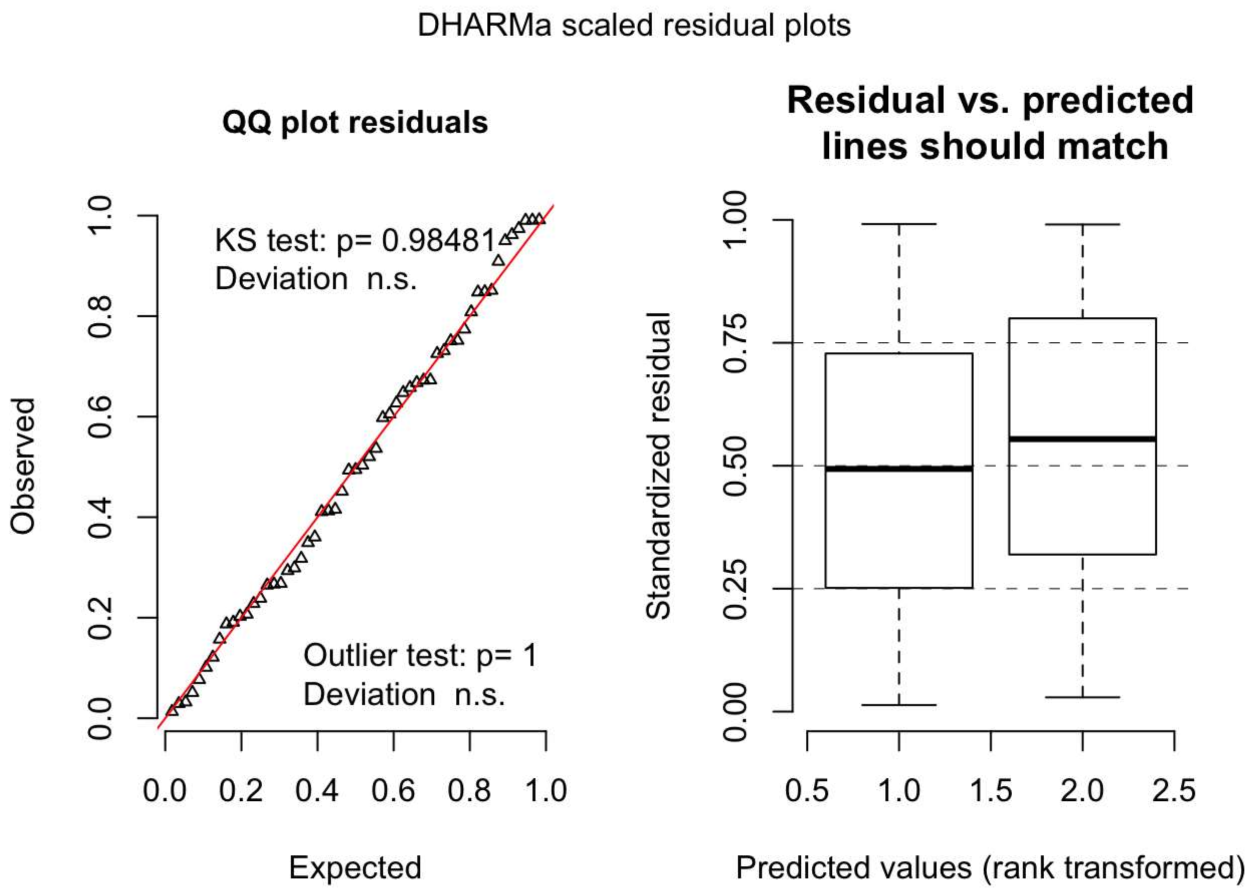


Test for temporal autocorrelation

```
resYear <- recalculateResiduals(res, group=tiger.cc$Year, aggregateBy = mean)
testTemporalAutocorrelation(resYear, time=unique(tiger.cc$Year))
```

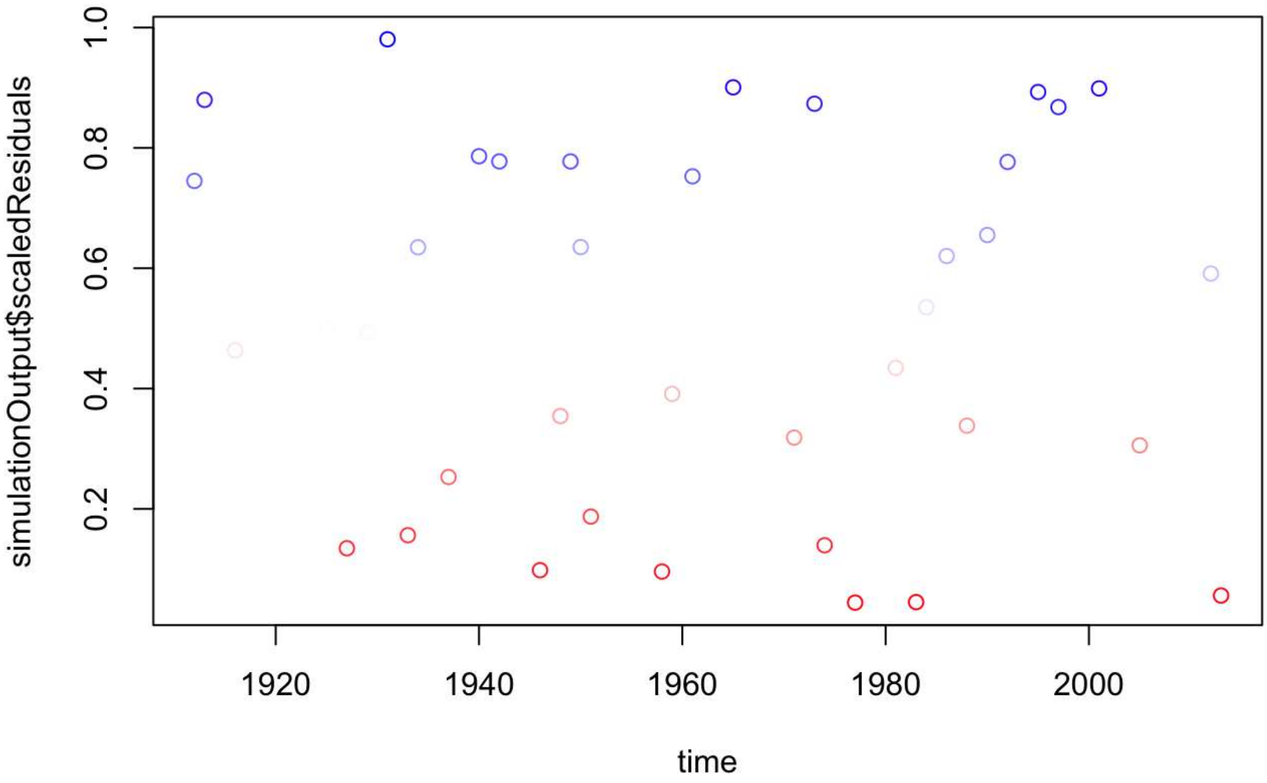


```
##
##  Durbin-Watson test
##
## data:  simulationOutput$scaledResiduals ~ 1
## DW = 1.8071, p-value = 0.5481
## alternative hypothesis: true autocorrelation is not 0
```

### Test for overdispersion

```
testDispersion(res)
```

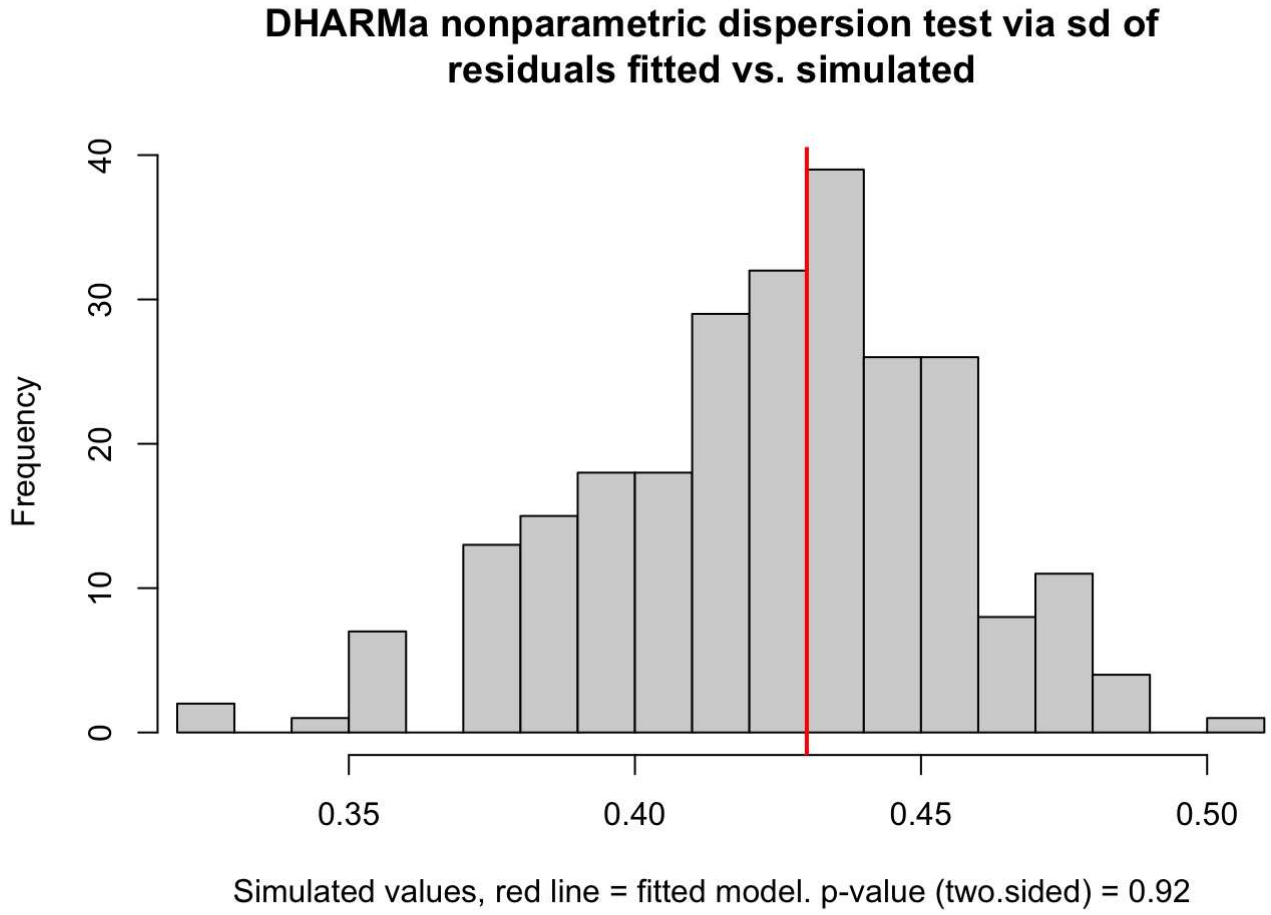


```
##
##  DHARMa nonparametric dispersion test via sd of residuals fitted vs.
##  simulated
##
## data:  simulationOutput
## ratioObsSim = 1.0168, p-value = 0.92
## alternative hypothesis: two.sided
```

## Test for zero inflation

```
testZeroInflation(res)
```

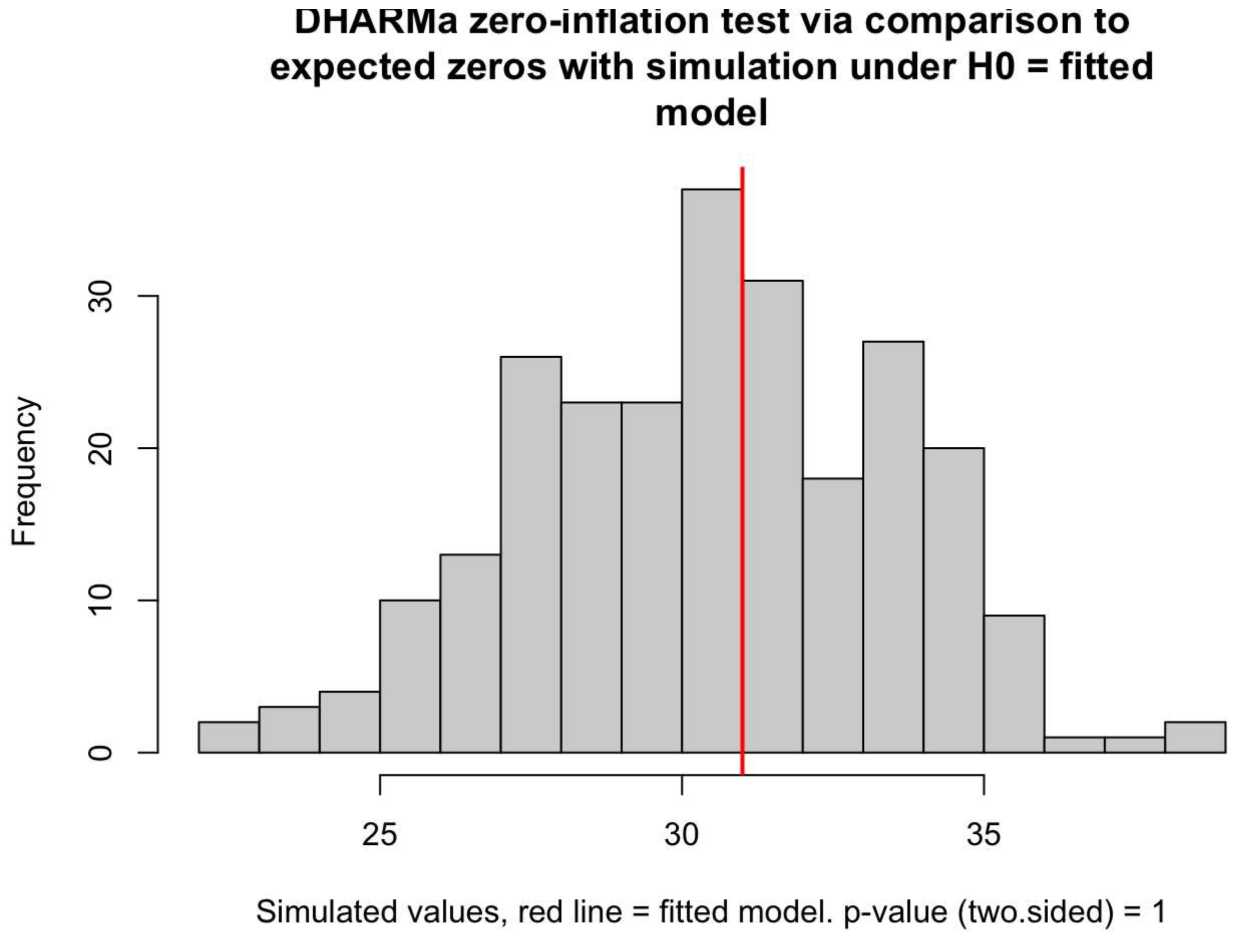


```
##
##  DHARMa zero-inflation test via comparison to expected zeros with
##  simulation under H0 = fitted model
##
## data:  simulationOutput
## ratioObsSim = 1.0012, p-value = 1
## alternative hypothesis: two.sided
```

# Plot results

Predict means and standard errors from model

```
nd <- data.frame(Activity = levels(tiger.cc$Activity))
pred <- predict(m9, nd, se.fit=T)
nd$Survival <- inv.logit(pred$fit)

nd$SE_upper <- inv.logit(pred$fit + pred$se.fit)
nd$SE_lower <- inv.logit(pred$fit - pred$se.fit)
nd$Activity <- factor(nd$Activity, labels=c("In-water","On-water"))
nd
```

```
##   Activity  Survival  SE_upper  SE_lower
## 1 In-water 0.3023256 0.3765543 0.2371630
## 2 On-water 0.9166667 0.9690025 0.7946897
```

Plots model predictions

```
ggplot(nd, aes(x=Activity, y= Survival)) + geom_bar(stat="identity") +
geom_linerange(aes(ymax=SE_upper, ymin=SE_lower)) +
ylab("Probability of Survival") +
xlab("Victim activity") +
ggtitle("Tiger Sharks")
```

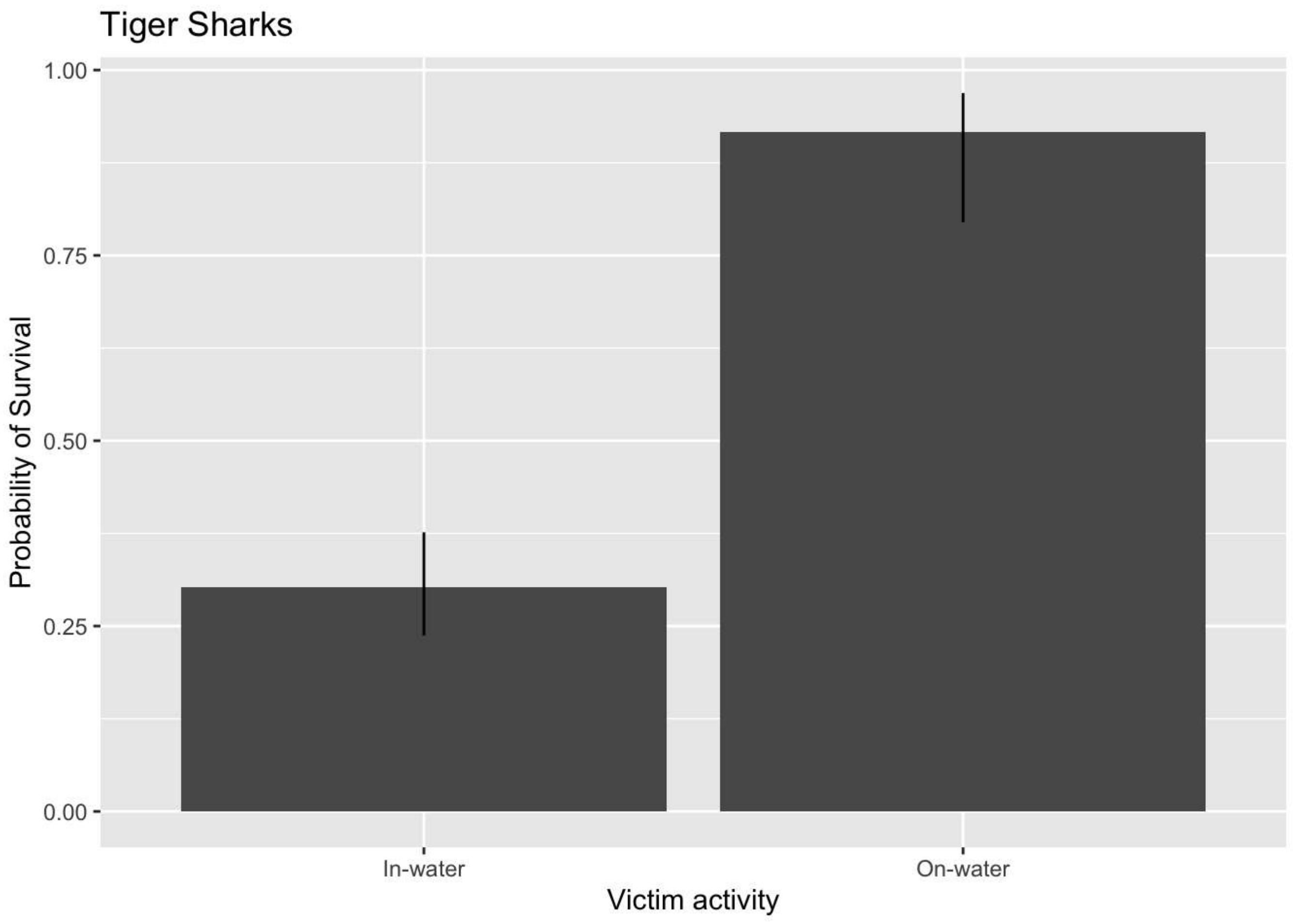

Supplement: Supplementary file 2 — Supplementary Information 2. [file 41598_2022_16950_MOESM2_ESM.pdf]
