# Supplementary material for: Increased shark bite survivability revealed by two centuries of Australian records
Source: Sci Rep. 2022 Aug 19;12:14121. doi: 10.1038/s41598-022-16950-5 (PMC9391475; doi:10.1038/s41598-022-16950-5)

WHITE SHARKS

- Load data and libraries

```
dd <- read.csv("SharkDeaths.csv")
library(DHARMa)
library(ggplot2)
library(GGally)
library(boot)
library(visdat)
```

- remove TimeOfDay
- subset for the species
- add Survival

```
dd$TimeOfDay <- NULL
dd$Survival <- 1 - dd$Death
white <- dd[dd$Species=="WS",]
```

- inspect missing data and correlations between predictor variables

```
vis_miss(white)
```

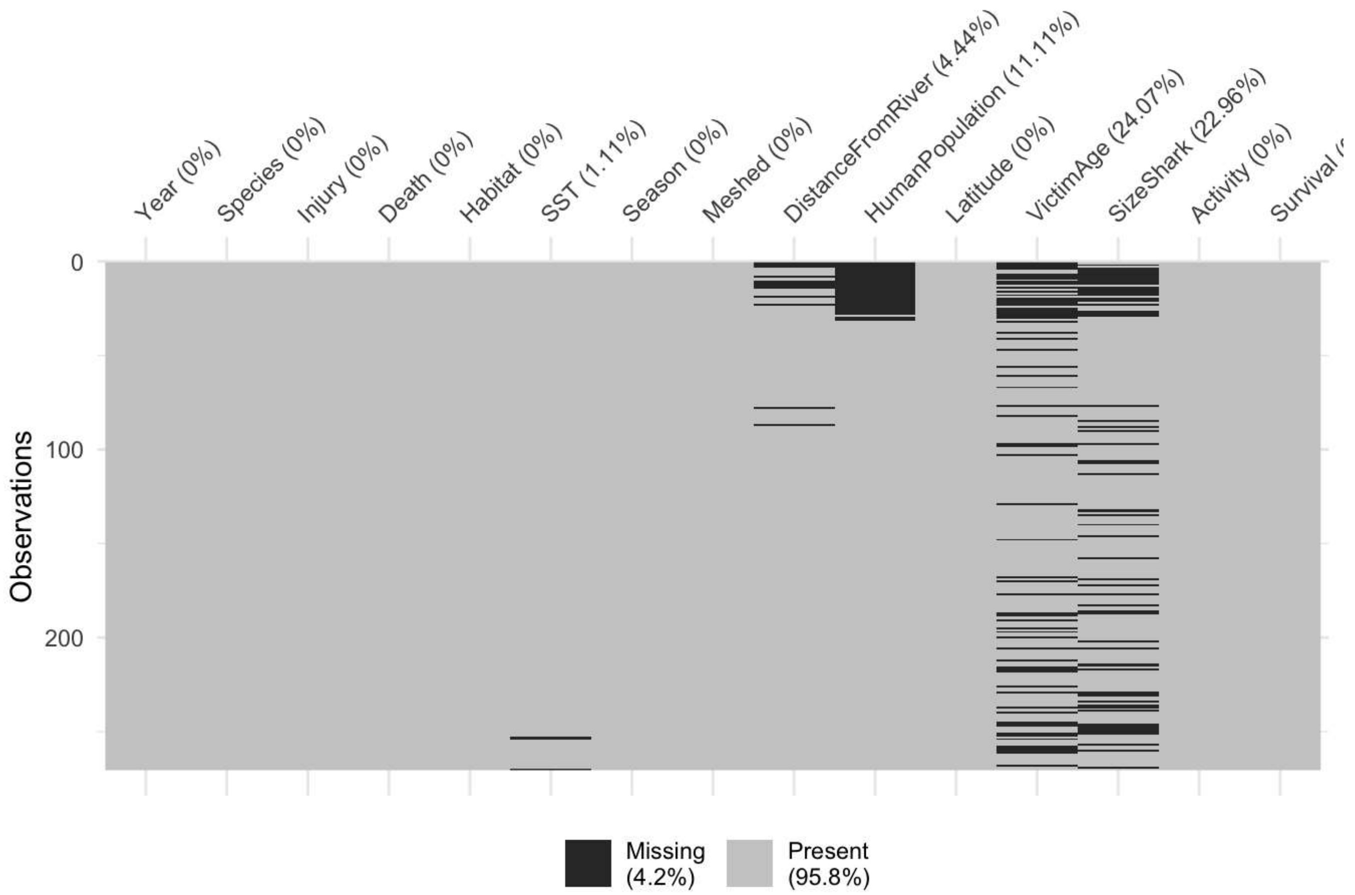


```
numericPredictors <- names(white)[names(white) %in%
                        c("Habitat","Species","Season","Activity") == F]
ggpairs(white[,numericPredictors])
```

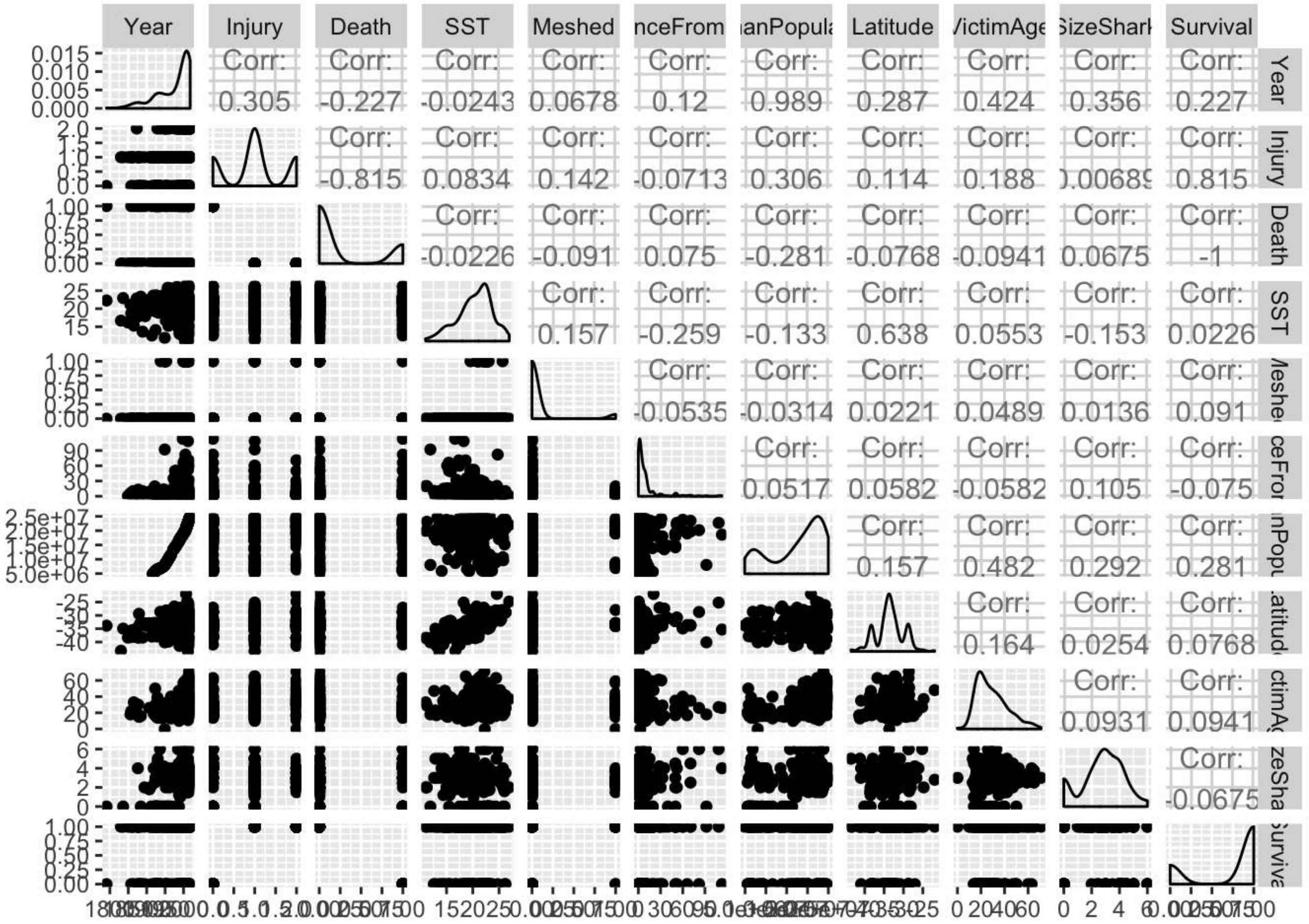

- Remove Human Population (correlated with Year)
- Remove Injury
- SST and latitude are correlated (>0.6). Remove SST.

```
white$ HumanPopulation <- NULL
white$ Injury <- NULL
white$ SST <- NULL

numericPredictors <- names(white)[names(white) %in%
                        c("Habitat","Species","Season","Activity") == F]
ggpairs(white[,numericPredictors])
```

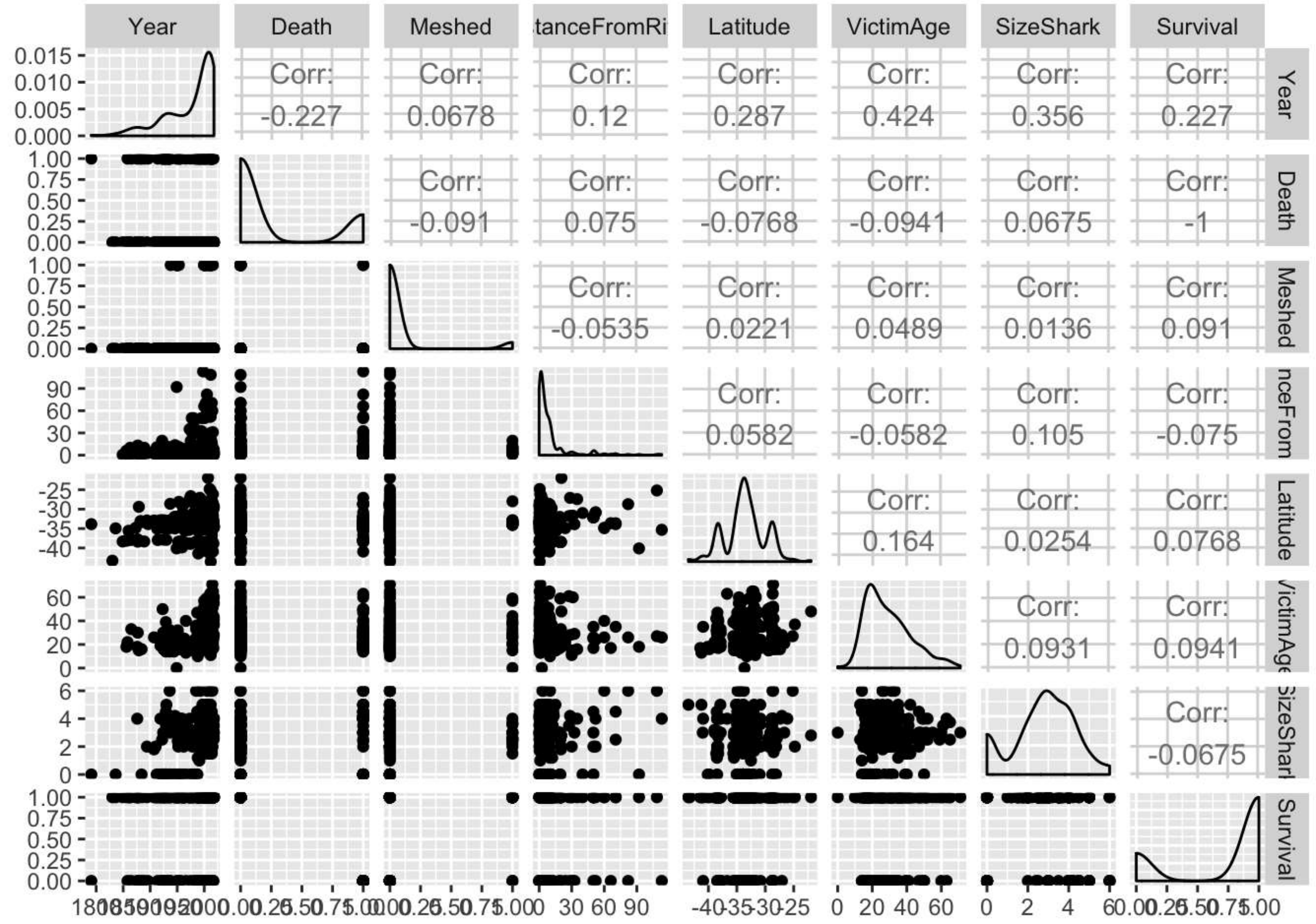


```
vis_miss(white)
```

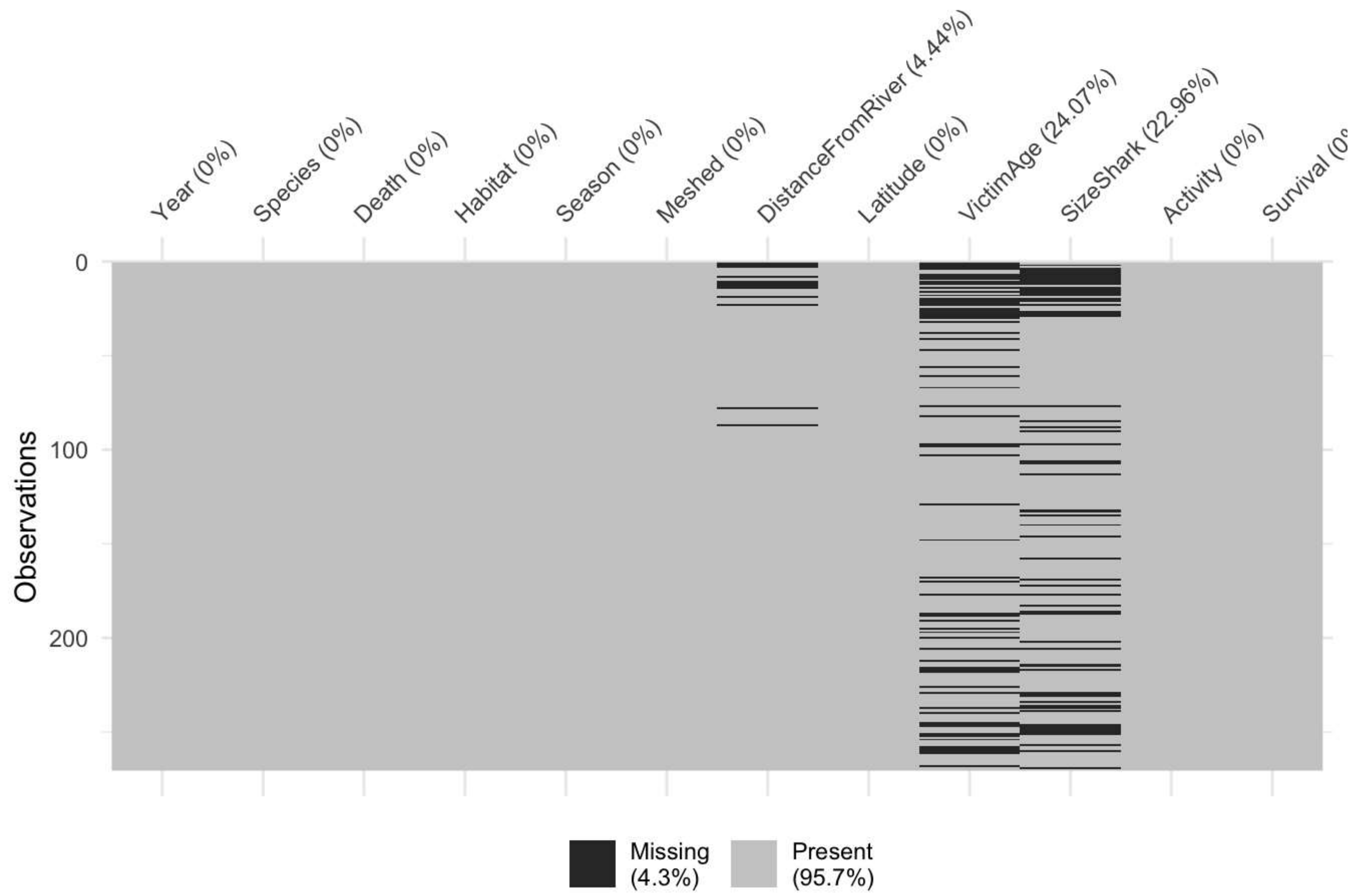

- No correlations > 0.6

- Missing data in 3 predictors. Approach used for missing data was listwise deletion, i.e.�only use complete cases for modelling

MODELLING

Use only complete cases

```
white.cc <- na.omit(white)
```

START MODEL: Survival ~ Year + Habitat + Season + Meshed + DistanceFromRiver + Latitude + Activity + VictimAge + SizeShark

```
m1 <- glm(Survival ~ Year + Habitat + Season + Meshed + DistanceFromRiver + Latitude + Activi
ty + VictimAge + SizeShark, data = white.cc, family=binomial)

drop1(m1, test="Chisq")
```

```
## Single term deletions
##
## Model:
## Survival ~ Year + Habitat + Season + Meshed + DistanceFromRiver +
##     Latitude + Activity + VictimAge + SizeShark
##                   Df Deviance    AIC     LRT  Pr(>Chi)
## <none>                 151.79 185.79
## Year               1   151.94 183.94  0.1538    0.6950
## Habitat            6   156.84 178.84  5.0532    0.5370
## Season             3   152.05 180.05  0.2612    0.9671
## Meshed             1   152.94 184.94  1.1533    0.2829
## DistanceFromRiver  1   152.52 184.52  0.7332    0.3918
## Latitude           1   151.82 183.82  0.0290    0.8648
## Activity           1   168.28 200.28 16.4923 4.885e-05 ***
## VictimAge          1   151.79 183.79  0.0001    0.9908
## SizeShark          1   153.79 185.79  2.0051    0.1568
## ---
## Signif. codes:  0 '***' 0.001 '**' 0.01 '*' 0.05 '.' 0.1 ' ' 1
```

Remove VictimAge

```
m2 <- update(m1, .~. - VictimAge)
drop1(m2, test="Chisq")
```

```
## Single term deletions
##
## Model:
## Survival ~ Year + Habitat + Season + Meshed + DistanceFromRiver +
##     Latitude + Activity + SizeShark
##                   Df Deviance    AIC     LRT  Pr(>Chi)
## <none>                 151.79 183.79
## Year               1   152.00 182.00  0.2047    0.6509
## Habitat            6   156.85 176.85  5.0561    0.5366
## Season             3   152.05 178.05  0.2615    0.9671
## Meshed             1   152.94 182.94  1.1540    0.2827
## DistanceFromRiver  1   152.54 182.54  0.7490    0.3868
## Latitude           1   151.82 181.82  0.0289    0.8651
## Activity           1   169.06 199.06 17.2721 3.239e-05 ***
## SizeShark          1   153.85 183.85  2.0638    0.1508
## ---
## Signif. codes:  0 '***' 0.001 '**' 0.01 '*' 0.05 '.' 0.1 ' ' 1
```

Remove Habitat

```
m3 <- update(m2, .~. - Habitat)
drop1(m3, test="Chisq")
```

```
## Single term deletions
##
## Model:
## Survival ~ Year + Season + Meshed + DistanceFromRiver + Latitude +
##     Activity + SizeShark
##                   Df Deviance    AIC     LRT  Pr(>Chi)
## <none>                 156.85 176.85
## Year               1   157.58 175.58  0.7324    0.3921
## Season             3   157.69 171.69  0.8420    0.8394
## Meshed             1   157.90 175.90  1.0485    0.3058
## DistanceFromRiver  1   157.13 175.13  0.2821    0.5953
## Latitude           1   157.04 175.04  0.1925    0.6608
## Activity           1   174.87 192.87 18.0247 2.181e-05 ***
## SizeShark          1   159.42 177.42  2.5762    0.1085
## ---
## Signif. codes:  0 '***' 0.001 '**' 0.01 '*' 0.05 '.' 0.1 ' ' 1
```

Remove Season

```
m4 <- update(m3, .~. - Season)
drop1(m4, test="Chisq")
```

```
## Single term deletions
##
## Model:
## Survival ~ Year + Meshed + DistanceFromRiver + Latitude + Activity +
##     SizeShark
##                   Df Deviance    AIC     LRT  Pr(>Chi)
## <none>                 157.69 171.69
## Year               1   158.66 170.66  0.9693    0.3249
## Meshed             1   158.74 170.74  1.0532    0.3048
## DistanceFromRiver  1   158.25 170.25  0.5601    0.4542
## Latitude           1   157.85 169.85  0.1642    0.6853
## Activity           1   175.65 187.65 17.9619 2.254e-05 ***
## SizeShark          1   160.15 172.15  2.4646    0.1164
## ---
## Signif. codes:  0 '***' 0.001 '**' 0.01 '*' 0.05 '.' 0.1 ' ' 1
```

Remove Latitude

```
m5 <- update(m4, .~. - Latitude)
drop1(m5, test="Chisq")
```

```
## Single term deletions
##
## Model:
## Survival ~ Year + Meshed + DistanceFromRiver + Activity + SizeShark
##                   Df Deviance    AIC     LRT  Pr(>Chi)
## <none>                 157.85 169.85
## Year               1   158.83 168.83  0.9770    0.3230
## Meshed             1   158.85 168.85  1.0007    0.3171
## DistanceFromRiver  1   158.44 168.44  0.5825    0.4453
## Activity           1   175.74 185.74 17.8894 2.341e-05 ***
## SizeShark          1   160.35 170.35  2.5027    0.1137
## ---
## Signif. codes:  0 '***' 0.001 '**' 0.01 '*' 0.05 '.' 0.1 ' ' 1
```

Remove DistanceFromRiver

```
m6 <- update(m5, .~. -DistanceFromRiver)
drop1(m6, test="Chisq")
```

```
## Single term deletions
##
## Model:
## Survival ~ Year + Meshed + Activity + SizeShark
##           Df Deviance    AIC     LRT  Pr(>Chi)
## <none>         158.44 168.44
## Year       1   159.28 167.28  0.8446   0.35807
## Meshed     1   159.53 167.53  1.0904   0.29638
## Activity   1   176.50 184.50 18.0646 2.135e-05 ***
## SizeShark  1   161.17 169.17  2.7380   0.09799 .
## ---
## Signif. codes:  0 '***' 0.001 '**' 0.01 '*' 0.05 '.' 0.1 ' ' 1
```

Remove Year

```
m7 <- update(m6, .~. -Year)
drop1(m7, test="Chisq")
```

```
## Single term deletions
##
## Model:
## Survival ~ Meshed + Activity + SizeShark
##           Df Deviance    AIC    LRT  Pr(>Chi)
## <none>         159.28 167.28
## Meshed     1   160.37 166.37  1.086    0.2973
## Activity   1   195.27 201.27 35.986 1.987e-09 ***
## SizeShark  1   161.23 167.23  1.951    0.1624
## ---
## Signif. codes:  0 '***' 0.001 '**' 0.01 '*' 0.05 '.' 0.1 ' ' 1
```

Remove Meshed

```
m8 <-update(m7, .~. -Meshed)
drop1(m8, test="Chisq")
```

```
## Single term deletions
##
## Model:
## Survival ~ Activity + SizeShark
##           Df Deviance    AIC    LRT  Pr(>Chi)
## <none>         160.37 166.37
## Activity   1   196.80 200.80 36.431 1.582e-09 ***
## SizeShark  1   162.30 166.30  1.934    0.1644
## ---
## Signif. codes:  0 '***' 0.001 '**' 0.01 '*' 0.05 '.' 0.1 ' ' 1
```

Remove SizeShark

```
m9 <- update(m8, .~. -SizeShark)
drop1(m9, test="Chisq")
```

```
## Single term deletions
##
## Model:
## Survival ~ Activity
##          Df Deviance   AIC    LRT  Pr(>Chi)
## <none>         162.3 166.3
## Activity  1    198.5 200.5 36.199 1.782e-09 ***
## ---
## Signif. codes:  0 '***' 0.001 '**' 0.01 '*' 0.05 '.' 0.1 ' ' 1
```

Final model: Survival ~ Activity

```
summary(m9)
```

```
##
## Call:
## glm(formula = Survival ~ Activity, family = binomial, data = white.cc)
##
## Deviance Residuals:
##     Min       1Q   Median       3Q      Max
## -2.0736  -1.0987   0.4977   0.4977   1.2582
##
## Coefficients:
##             Estimate Std. Error z value Pr(>|z|)
## (Intercept)  -0.1881     0.2511  -0.749    0.454
## ActivityON    2.2140     0.3967   5.581 2.39e-08 ***
## ---
## Signif. codes:  0 '***' 0.001 '**' 0.01 '*' 0.05 '.' 0.1 ' ' 1
##
## (Dispersion parameter for binomial family taken to be 1)
##
##     Null deviance: 198.5  on 166  degrees of freedom
## Residual deviance: 162.3  on 165  degrees of freedom
## AIC: 166.3
##
## Number of Fisher Scoring iterations: 4
```

Diagnostics

```
res <- simulateResiduals(fittedModel = m9, n = 250)
plot(res)
```

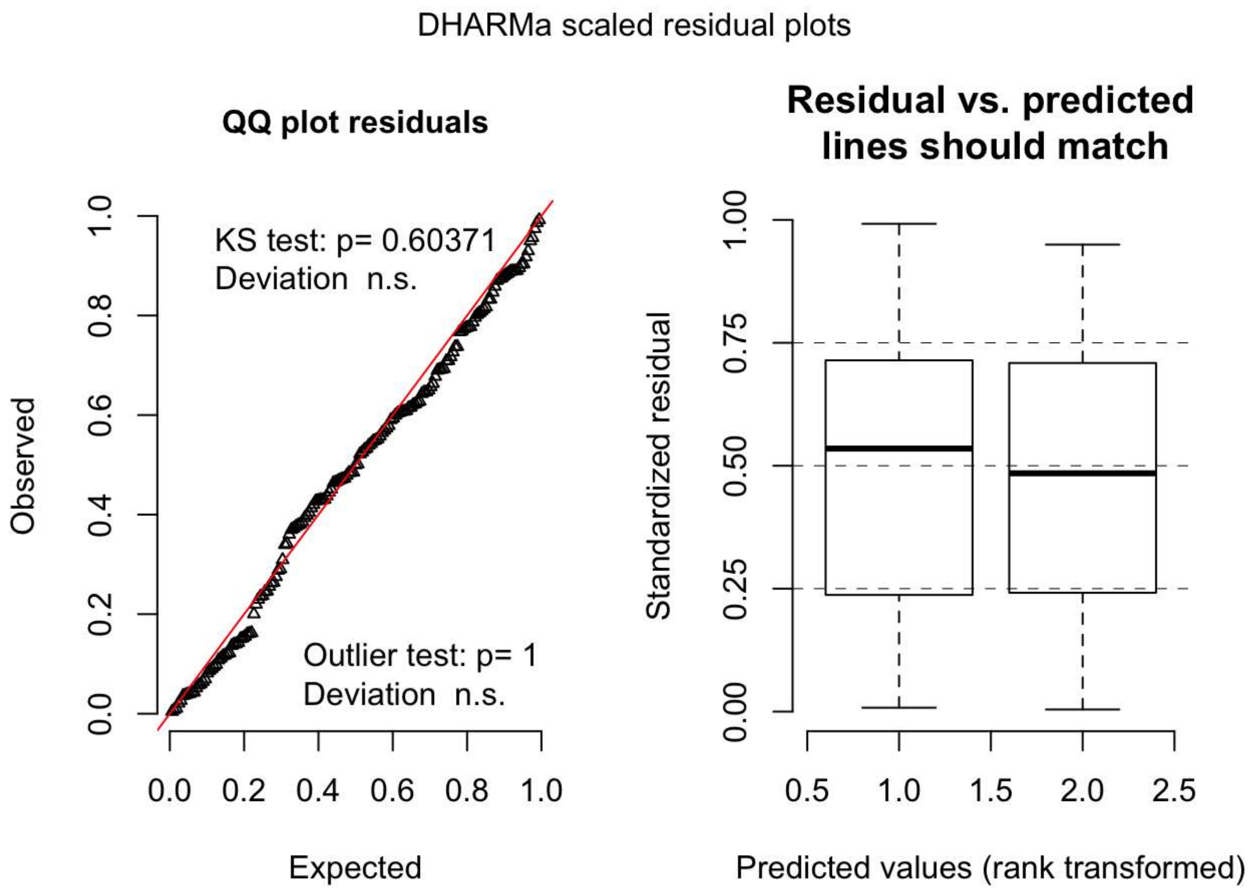

Test for temporal autocorrelation

```
resYear <- recalculateResiduals(res, group=white.cc$Year, aggregateBy = mean)
testTemporalAutocorrelation(resYear, time=unique(white.cc$Year))
```

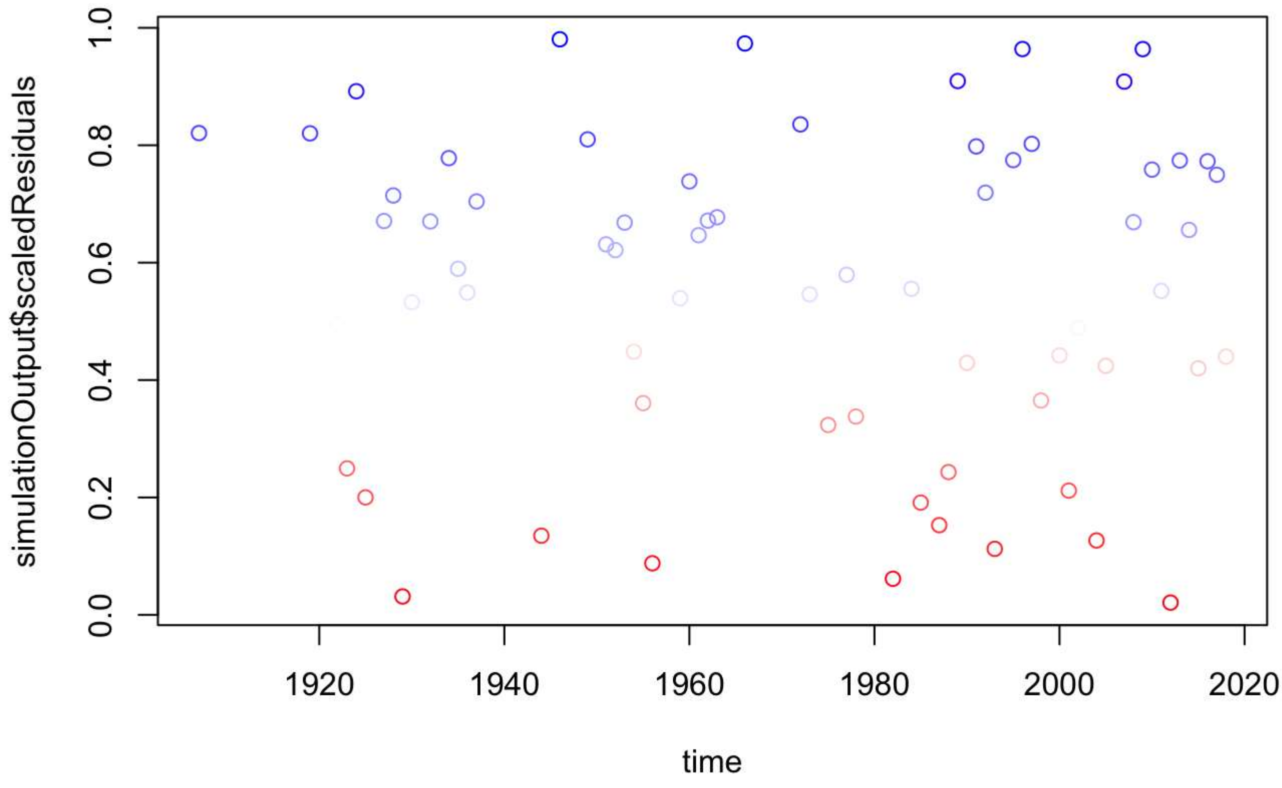


```
##
##  Durbin-Watson test
##
## data:  simulationOutput$scaledResiduals ~ 1
## DW = 1.8039, p-value = 0.4192
## alternative hypothesis: true autocorrelation is not 0
```

Test for overdispersion

```
testDispersion(res)
```

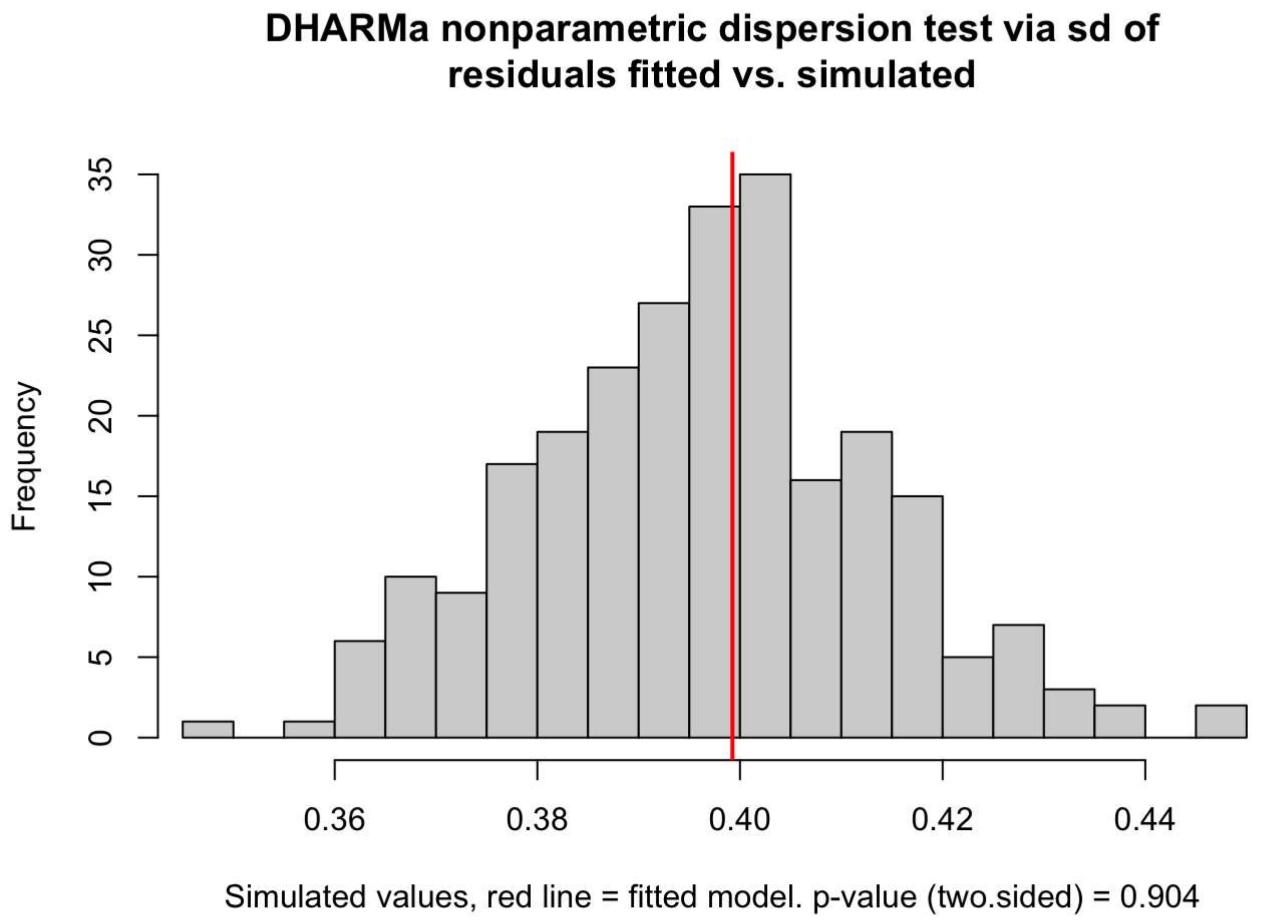


```
##
##  DHARMa nonparametric dispersion test via sd of residuals fitted vs.
##  simulated
##
## data:  simulationOutput
## ratioObsSim = 1.0073, p-value = 0.904
## alternative hypothesis: two.sided
```

Test for zero inflation

```
testZeroInflation(res)
```

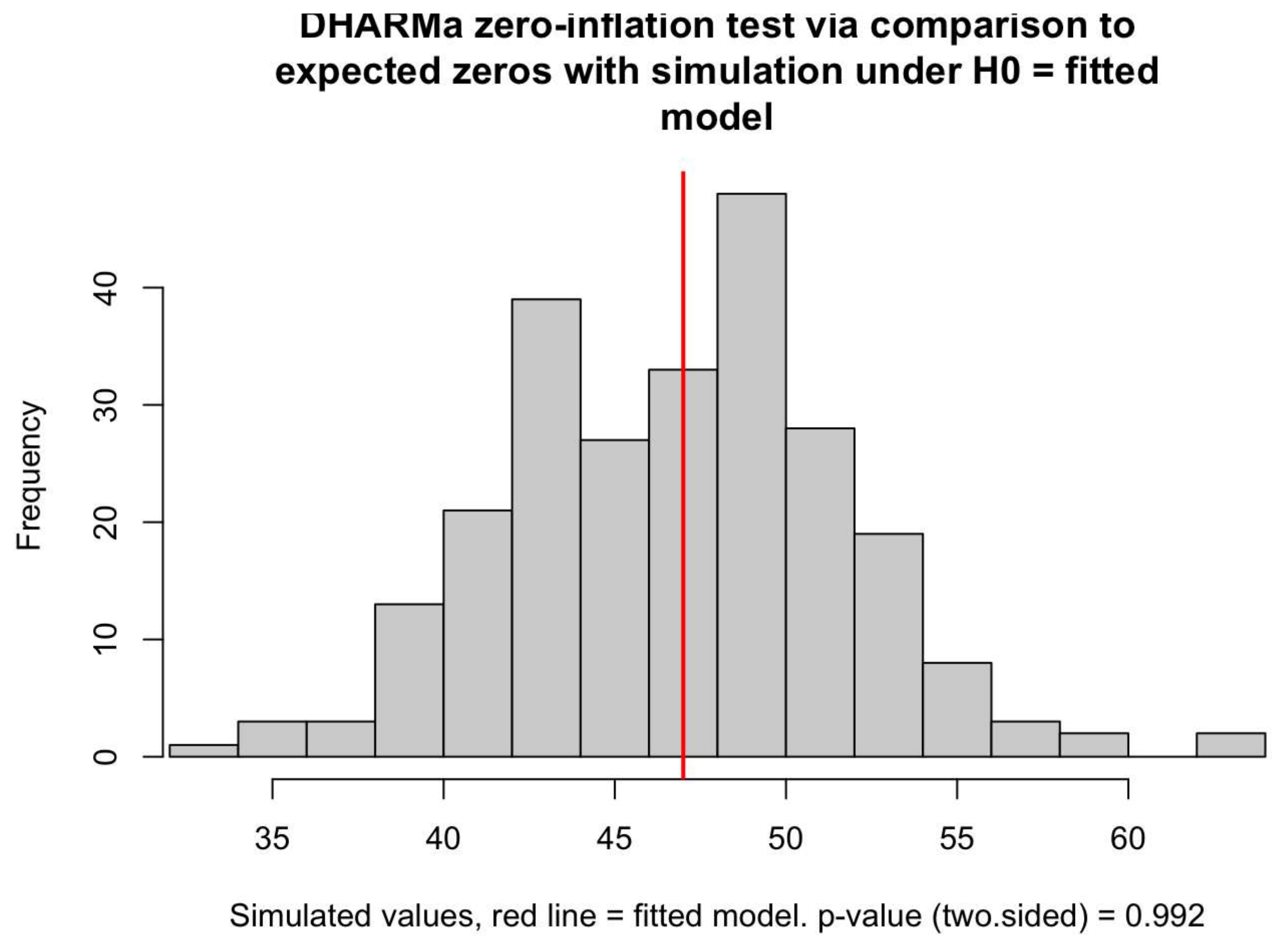


```
##
##  DHARMa zero-inflation test via comparison to expected zeros with
##  simulation under H0 = fitted model
##
## data:  simulationOutput
## ratioObsSim = 0.9945, p-value = 0.992
## alternative hypothesis: two.sided
```

Plot results

Predict means and standard errors from model

```
nd <- data.frame(Activity = levels(white.cc$Activity))
pred <- predict(m9, nd, se.fit=T)
nd$Survival <- inv.logit(pred$fit)

nd$SE_upper <- inv.logit(pred$fit + pred$se.fit)
nd$SE_lower <- inv.logit(pred$fit - pred$se.fit)
nd$Activity <- factor(nd$Activity, labels=c("In-water","On-water"))
nd
```

```
##   Activity  Survival  SE_upper  SE_lower
## 1 In-water 0.4531250 0.5157582 0.3919416
## 2 On-water 0.8834951 0.9115790 0.8479790
```

Plots model predictions

```
ggplot(nd, aes(x=Activity, y= Survival)) + geom_bar(stat="identity") +
geom_linerange(aes(ymax=SE_upper, ymin=SE_lower)) +
ylab("Probability of Survival") +
xlab("Victim activity") +
ggtitle("White Sharks")
```

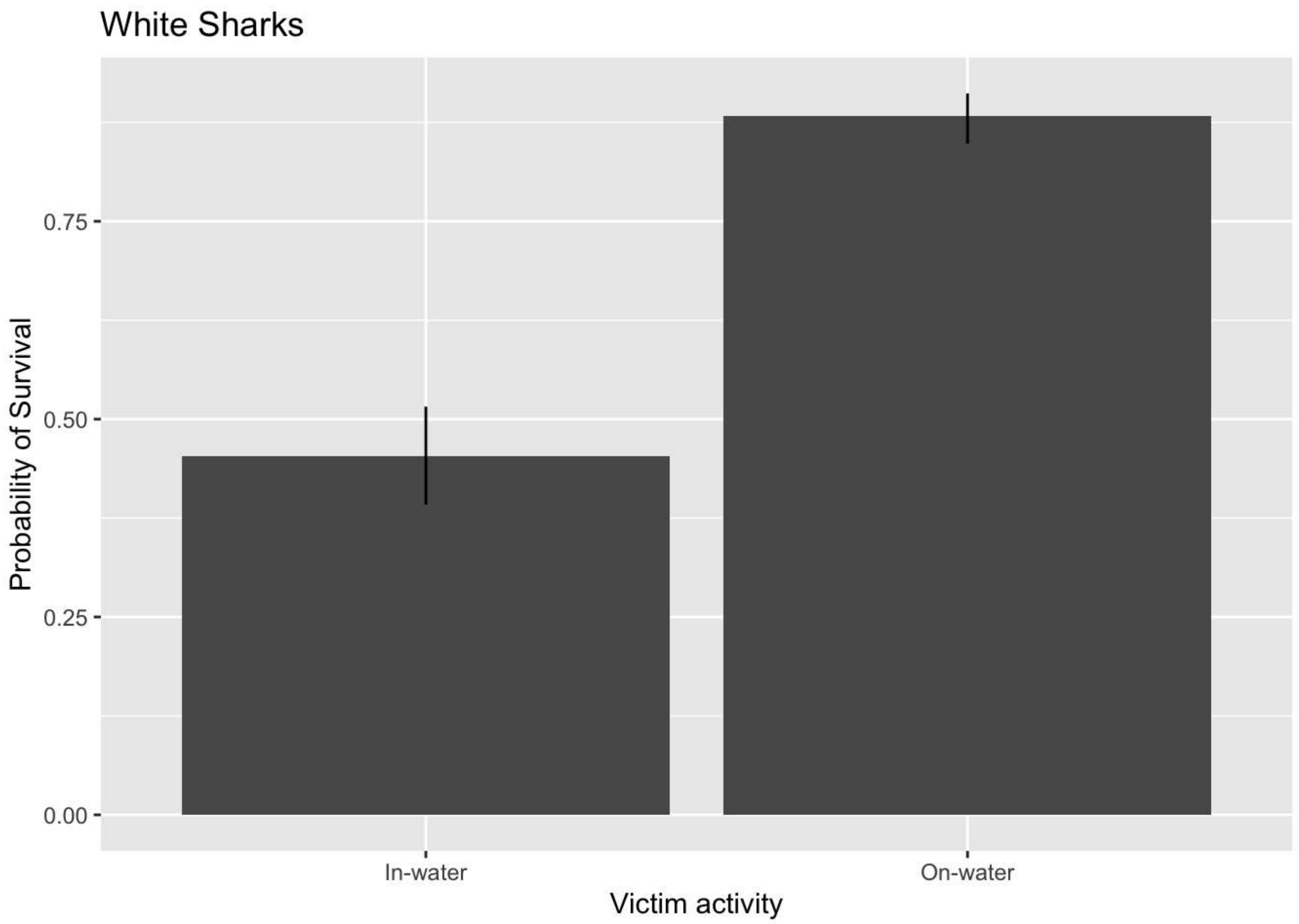

Supplement: Supplementary file 3 — Supplementary Information 3. [file 41598_2022_16950_MOESM3_ESM.pdf]
